# Exploration of Bioactive Umami Peptides from Wheat Gluten: Umami Mechanism, Antioxidant Activity, and Potential Disease Target Sites

**DOI:** 10.3390/foods13233805

**Published:** 2024-11-26

**Authors:** Haowen Chen, Huiyan Zhao, Cuiling Li, Chunxia Zhou, Jianxu Chen, Wenjie Xu, Guili Jiang, Jingjing Guan, Zhuorong Du, Donghui Luo

**Affiliations:** 1 College of Food Science and Technology, College of Food Science and Engineering, Guangdong Ocean University, Zhanjiang 524088, China; 2112203106@stu.gdou.edu.cn (H.C.); zhaohuiyan@gdou.edu.cn (H.Z.); 19076471213@163.com (C.L.); chunxia.zhou@163.com (C.Z.); xuwenjie@gdou.edu.cn (W.X.); jgl@gdou.edu.cn (G.J.); gjj@gdou.edu.cn (J.G.); duzhuorong@gdou.edu.cn (Z.D.); 2 Branch of Chemistry and Chemical Engineering Guangdong Laboratory (Hanjiang Laboratory), Chaozhou 521000, China; 3 Guangdong Mei Wei Yuan Flavours Co., Ltd., Yangjiang 529500, China; muyuzikui@163.com

**Keywords:** wheat gluten, umami peptide, molecular dynamics simulations, antioxidant, network pharmacology

## Abstract

Umami peptides have the ability to enhance food flavours and have potential health benefits. The objective of this study was to conduct a comprehensive investigation into the umami intensity, taste mechanism, and antioxidant activity of six umami peptides derived from wheat gluten hydrolysates (WGHs) and fermented WGHs. The e-tongue analysis demonstrated that the peptides exhibited a direct proportionality in terms of umami value and concentration, and were capable of enhancing the umami of commercially available condiments. The molecular dynamics simulations demonstrated that the peptides interacted with T1R1/T1R3 receptors via hydrogen bonds, hydrophobic interactions, ionic interactions, and water bridges, thereby producing umami. Furthermore, the DPPH, ABTS, hydroxyl radical-scavenging, and FRAP assays demonstrated that the six peptides exhibited antioxidant activity in vitro. Ultimately, the network pharmacology and molecular docking results indicated that AKT1, JUN, and CASP3 may serve as the core targets for the peptides in the treatment of oxidative diseases. In conclusion, this work offers novel insights into the use of bioactive umami peptides, emphasising their prospective applications in the food and health supplement industries.

## 1. Introduction

Umami, which is recognised as the one of the five basic tastes, plays a pivotal role in the perception of food taste. Umami compounds possess two distinctive characteristics: synergistic effects and interactions with other flavours [1]. A number of natural umami substances have been identified in a range of foods, with fermented foods being a notable source. These foods contain bioactive compounds that contribute significantly to umami. The interest in peptides associated with umami tastes has grown considerably in recent times, becoming a prominent area of research within the food industry. This is largely driven by the increasing demand for tasty and natural food ingredients. Enzymatic hydrolysis or fermentation of plant proteins has unequivocally resulted in the creation of numerous peptides that contribute to taste [2]. Commercial wheat gluten (WG) is a potential raw material for high-quality presentation bases due to its high protein content and low cost. Its primary structure, which is rich in umami peptide sequences, makes it an ideal candidate for this purpose [3]. Six umami peptides (QQLPQFEE, LSFE, EELR, YTCE, YTTD, and EEDQ) were identified from the enzymatic fermentation products of WG in the early stage of our research [4], but their umami mechanisms and active functions have not yet been fully explored. Moreover, while the ability of umami peptides to enhance taste has been demonstrated in MSG solutions and salt solutions, their efficacy in actual food systems remains to be seen. Currently, molecular docking is mainly used in virtual studies to analyse the interaction of peptides with specific receptors, a process that is considered to be static. As more and more umami peptides are being discovered, molecular dynamics (MD) simulations have become a highly regarded technique in the field of molecular modelling. They offer a solution to the challenge of conformational alterations in proteins and small molecules that are not incorporated into molecular docking procedures. More importantly, MD simulations can elucidate the mechanism of flavour generation from a dynamic perspective [5]. The search for biologically active umami peptides is still an ongoing process.

Peptides are frequently employed as food additives with the objective of enhancing gustatory sensations. They are regarded as functional actives with a range of biological activities (antioxidant activity, lowering of blood pressure, immune regulation, and more) [6]. The antioxidant activity of peptides is of particular importance. This activity can protect the body from the damaging effects of free radicals and, at the same time, it can slow down the progression of chronic diseases [7]. Oxidative damage is a negative phenomenon that occurs when there is an imbalance between reactive oxygen species (ROS) and antioxidants. This physiological state is linked to a wide range of disorders, which makes it an intriguing area of study [8]. Peptides have the capacity to stimulate the Keap1-Nrf2 pathway. This pathway has already been demonstrated to be the fundamental mechanism in protecting the body from oxidative damage. Consequently, stimulation of this pathway results in diminished oxidative damage from external irritation. In order to reduce oxidative damage, researchers have shifted their attention from synthetic antioxidants, which may have adverse effects on the digestive system, to non-toxic natural antioxidants [2]. This transition underscores the significance of antioxidant peptides in the battle against free radicals. Recent studies have revealed that certain peptides serve a dual function: taste enhancement and antioxidant properties. Umami peptides with molecular weights below 1 kDa extracted from pig bone proteins showed notable antioxidant efficacy in both ex vivo and in vivo trials [9]. Not coincidentally, the three umami peptides produced in douchi in the presence of microorganisms also have antioxidant characteristics [10]. The potential of umami peptides with antioxidant properties to serve as natural additives or preservatives for food materials is significant. By extending the shelf life of these foods and reducing food waste, they could have a beneficial impact on the food industry. Furthermore, their use as nutritional supplements or functional ingredients may be enhance functional properties while imparting a favourable taste to products [11]. It is known that there is a paucity of published studies examining the antioxidant activity of umami peptides derived from WGHs and fermented WGHs.

There is an increasing acceptance of the concept of “medicine and food homology” within the field of health. In accordance with the stipulations set forth in the Chinese Food Safety Law, the term “food” encompasses a comprehensive array of raw materials and products utilised for the purposes of nourishment and consumption. This definition can be extended to encompass ingredients traditionally used as both food and herbal ingredients. However, items utilised for therapeutic purposes are excluded from this definition. The chemical components of food are highly diverse, paralleling the complexity observed in traditional Chinese medicine. Nevertheless, the principal purpose of food is to satisfy the body’s nutritional requirements, rather than to exert healing actions. The relatively low biological activity of these substances presents a challenge when attempting to study them from the perspective of traditional pharmacological modelling. In light of the increasing popularity of the concept of functional foods, the research on bioactive foods is gradually shifting towards a more diversified direction. Consequently, the conventional research paradigm of ‘single component–single target’ is insufficient to address the evolving demands in this domain. The emergence of genomics and big data has catalysed the development of interdisciplinary fields such as bioinformatics and systems biology, encouraging researchers around the world to investigate functional foods through a lens that emphasises network construction and molecular-level analyses [12,13]. It has served as a crucial instrument for elucidating the mechanisms of action and health benefits of functional components in food [14]. Researchers have employed network pharmacology to reveal the therapeutic potential of soy-fermented foods in the management of lung cancer [15]. In addition, network pharmacology and molecular docking techniques have also been used to explore the potential application of Taxus chinensis fruit in combating Alzheimer’s disease [16]. The study of the hyperuricemia inhibitor from the food-based plant Perillae Folium has also received the help of network pharmacology [17]. It can be reasonably deduced that network pharmacology will facilitate the study of peptide bioactivity.

Umami peptides have the potential to become flavourings with health benefits, including antioxidant activity. Nevertheless, there is a paucity of studies that concurrently investigated the potential antioxidant effects of umami peptides. Due to the multitude and diversity of polypeptide targets, their mechanisms of action are inherently complex. In view of the resemblance between the concept of ‘multi-component–multi-target’ features of food and Chinese medicine, network pharmacology was hypothesised to be applicable to the study of the mechanism of the medicinal functions of peptides [18]. In this study, an electronic tongue (e-tongue) was used to verified the umami activities of six peptides identified from WGHs and fermented WGHs. Additionally, the umami presentation mechanisms of these peptides were explored through molecular dynamics simulations. However, the question of whether these six umami peptides possess antioxidant activity remained unresolved. Therefore, the objective of this research was to successfully apply the network pharmacology to study the interactions of umami peptides with potential antioxidant disease targets. Hopefully, our work will establish a foundation for the advancement of nutritional products designed to enhance health and tastes.

## 2. Materials and Methods

### 2.1. Materials and Chemicals

Umami synthetic peptides (QQLPQFEE, LSFE, EELR, YTCE, YTTD, and EEDQ, named QE-8, LE-4, ER-4, YE-4, YD-4, and EQ-4, respectively) were provided by Sangon Bioengineering Co. (Shanghai, China). Soy sauce (Haitian Flavouring and Food Co., Ltd., Foshan, China), chicken soup (Campbell Soup Swire Ltd., Hongkong, China), mushroom soup (Baili Foods Co., Zhaoqing, China), and fish soup (Knorr-Nahrmittel Ltd., Berne, Switzerland) were purchased from a market. 1,1-diphenyl-2-picrylhydrazyl (DPPH) and 2,2’-azino-bis (3-ethylbenzothiazoline-6-sulfonic acid) were purchased from Merck Life Science Co., Ltd. (Shanghai, China). The total antioxidant capacity assay kits for the FRAP method (Suzhou Grace Biotechnology Co., Ltd., Suzhou, China) and hydroxyl radical inhibition rate kits (Solarbio Science & Technology Co., Ltd., Beijing, China) were purchased from reagent companies.

### 2.2. Determination of Quantity–Effect Relationship of Umami Peptides

The synthetic peptide was prepared in distilled water (at concentrations of 2, 1, 0.5, 0.25, and 0.125 g/mL), and were injected for umami intensity determination using the INSENT-SA402B electronic tongue system (Intelligent Sensor Technology, Inc., Atsugi, Japan). A two-step cleaning method was employed for detection. This involved the utilisation of a cleaning solution (solution 1) for a duration of 90 s, a second cleaning solution (solution 2) for 120 s, a third cleaning solution (solution 3) for an additional 120 s, and finally, a conditioning solution. Subsequently, 30 s were allocated for sample selection, 30 s for cleaning using cleaning solution 4, 3 s for cleaning using cleaning solution 5, and 30 s for cleaning using cleaning solution 6 [1].

### 2.3. Determination of Application of Umami Peptides in Condiments

#### 2.3.1. Electronic Tongue

The peptides were dissolved and diluted to a concentration of 0.1 mg/mL with 10% soy sauce, chicken soup, mushroom soup, and fish soup to assess their umami-enhancing ability. The e-tongue analysis procedure was described in Section 2.2 [1].

#### 2.3.2. Sensory Evaluation

The peptides were dissolved and diluted to a concentration of 1 mg/mL with 10% soy sauce, chicken soup, mushroom soup, and fish soup to assess their umami-enhancing ability. Panellists (five females and five males, all with sensory training) were asked to rate the umami taste using a scale. Condiments at a concentration of 10% were the standard (5 points). All scorecards and mean values for all descriptors in three experiments were collected [1]. Prior to the start of the sensory assessment, all the participants gave informed consent to participate in this experiment.

### 2.4. Molecular Dynamics (MD) Simulation

The Desmond software (Version: Maestro_2021.1) was used to perform detailed simulations of protein–peptide complexes. To study how proteins and peptides interact, the OPLS2005 force field was used, and water molecules were represented using the TIP3P model. The protein–peptide complexes were put in a box with the right amount of chloride and sodium ions to balance the charge. Before starting the simulation, the steepest descent method was used to reduce the system’s energy over 50,000 steps, ensuring a stable start. After the equilibrium phase, we ran 100 nanosecond simulations to see how the protein–peptide complexes behaved when they were not bound. To record the simulation details, we performed readings at 10-picosecond intervals. The conditions were kept constant at 300 K and 1 bar.

### 2.5. Measurement of Antioxidant Activity

#### 2.5.1. DPPH

A DPPH working solution (ethanol preparation) was prepared at a concentration of 0.2 mmol, and the synthetic peptides were prepared at concentrations of 200, 100, 50, 25, 12.5 μg/mL. A 1 mL volume of the DPPH working solution was mixed with 1 mL of the sample, and the sample was incubated for 30 min at 25 °C. A 1:1 mixture of the DPPH working solution and anhydrous ethanol was used as a blank control. Vitamin C (Vc) was used as the positive control. The samples’ absorbance at 517 nm was measured using an ultraviolet spectrophotometer (Cintra1010-V4428, GBC Scientific Instruments, Inc., Melbourne, Australia) [19].

DPPH scavenging ability (%) = (1 − A1/A0) × 100(1)

where A1 is the absorbance of the sample and A0 is the absorbance of the blank control.

#### 2.5.2. ABTS

A total of 0.007 g of K2S2O8 and 0.0406 g of ABTS were added to 10 mL of distilled water and mixed well in equal volumes to prepare the ABTS working solution, which was left for 12–16 h and then diluted by 50 times. The reaction was carried out for 18 min by adding 0.1 mL of the synthetic peptide to 3 mL of the working solution. A 3 mL volume of the working solution was added to 0.1 m of distilled water to use as the blank control. Vc was used as the positive control. The samples’ absorbance at 734 nm was measured using an ultraviolet spectrophotometer (Cintra1010-V4428, GBC, Sydney, Australia) [19].

ABTS scavenging ability (%) = (1 − A1/A0) × 100(2)

where A1 is the absorbance of the sample and A0 is the absorbance of the blank control.

#### 2.5.3. FRAP

The reference kit method was used to detect FRAP. To prepare the working solution prior to utilisation, a mixture of diluted 2,4,6-tripyridyl-s-triazine (TPTZ), detection buffer, and a TPTZ solution was mixed in a volume ratio of 10:1:1. This working solution was then incubated at 37 °C. A sample of a 0.1 mg/mL peptide solution was introduced into 180 µL of the TPTZ solution within a zymography plate. Following a 30 min incubation period at 25 °C, the absorbance of the reaction mixture was recorded at 593 nm using a microplate reader (Bio-Rad, Hercules, CA, USA). The calculation equation was taken from [20].

#### 2.5.4. Hydroxyl Radicals

In accordance with the instructions provided in the assay kit, 200 μL of the sample was mixed thoroughly with 5 mL of a ferrous (2 mmol/L) sulphate solution and 5 mL of a hydrogen peroxide solution (6 mmol/L). A 2 mL volume of a salicylic acid solution (6 mmol/L) was added, and the absorbance value was immediately measured at 510 nm after thorough mixing. This value was designated as A0. Distilled water was used as the blank sample, and the absorbance value at 536 nm was designated as A1. Vc was used as the positive control. The following calculation equation was taken from [21]:

Hydroxyl radical scavenging ability (%) = [(A0 − A1)/A0] × 100(3)

where A1 is the absorbance of the sample and A0 is the absorbance of the blank control.

### 2.6. Construction of Network Pharmacology

#### 2.6.1. Prediction of Core Targets of Umami Peptides and Diseases

The peptides were entered into the SwissADME database in order to obtain the SMILES numbers, which were then imported into the SwissTarget Prediction database for the purpose of target prediction. The terms “oxidative damage” and “antioxidant” were used as search terms to identify disease-related genes in the OMIM (https://www.omim.org/, accessed on 21 May 2024), DisGeNET (https://www.disgenet.org/, accessed on 21 May 2024), and GeneCards (https://www.genecards.org/, accessed on 21 May 2024) databases. Following the collection of all targets, any duplicates were removed, and the target names were normalised. A Venn analysis was conducted to identify common targets between the disease and peptide targets. The aforementioned common targets were then imported into the STRING 11.5 database (https://cn.string-db.org/, accessed on 21 May 2024). Subsequently, the Cytoscape software (version 3.8.2, Cytoscape Consortium, San Francisco, CA, USA) was employed to identify the core targets. The selection of core targets was based on the degree, closeness, and betweenness values. Targets above the mean were considered potential core targets [22].

#### 2.6.2. Gene Ontology (GO) and Kyoto Encyclopedia of Genes and Genomes (KEGG) Analysis

To better understand how the core targets work and the metabolic pathways that they are involved in, they were imported into the David database (https://david.ncifcrf.gov/, accessed on 21 May 2024) for GO and KEGG analyses. The bioinformatics website was used to visualise the results and check the details (https://www.bioinformatics.com.cn/, accessed on 21 May 2024).

#### 2.6.3. Construction of Target-Pathway Network Maps

Target-pathway network maps were constructed using Cytoscape 3.8.2 software based on the key targets of peptide antioxidants and KEGG pathways, in combination with their interactions (top 20 signalling pathways sorted by *p*-value and 25 key targets).

### 2.7. Molecular Docking

The UniProt database was used to download the UniProt IDs of the core targets (https://www.uniprot.org/, accessed on 21 May 2024), and the PDB database was used to download the 3D structures of the receptor proteins (https://www.rcsb.org/, accessed on 21 May 2024). Chem3D was used to create the 3D structures of the peptides. AutoDock Vina 1.2.2, Discovery Studio 2019, and PyMOL 2.3.4 were used to implement the docking tasks [23]. Docking targets were obtained from the network pharmacological analyses. JUN (P05412), AKT1 (P31749), and CASP3 (P42574) were the receptors used in the molecular docking analysis.

### 2.8. Statistical Analysis

The Origin (Version: 2021pro) software (Origin Lab Co., Northampton, MA, USA) was used to produce the figures. The statistical analyses were conducted using SPSS 19 (SPSS Inc., Chicago, IL, USA). The data are presented as the mean ± standard deviation.

## 3. Results and Discussion

### 3.1. Quantity–Effect Relationship of Umami Peptides

**Figure 1:**
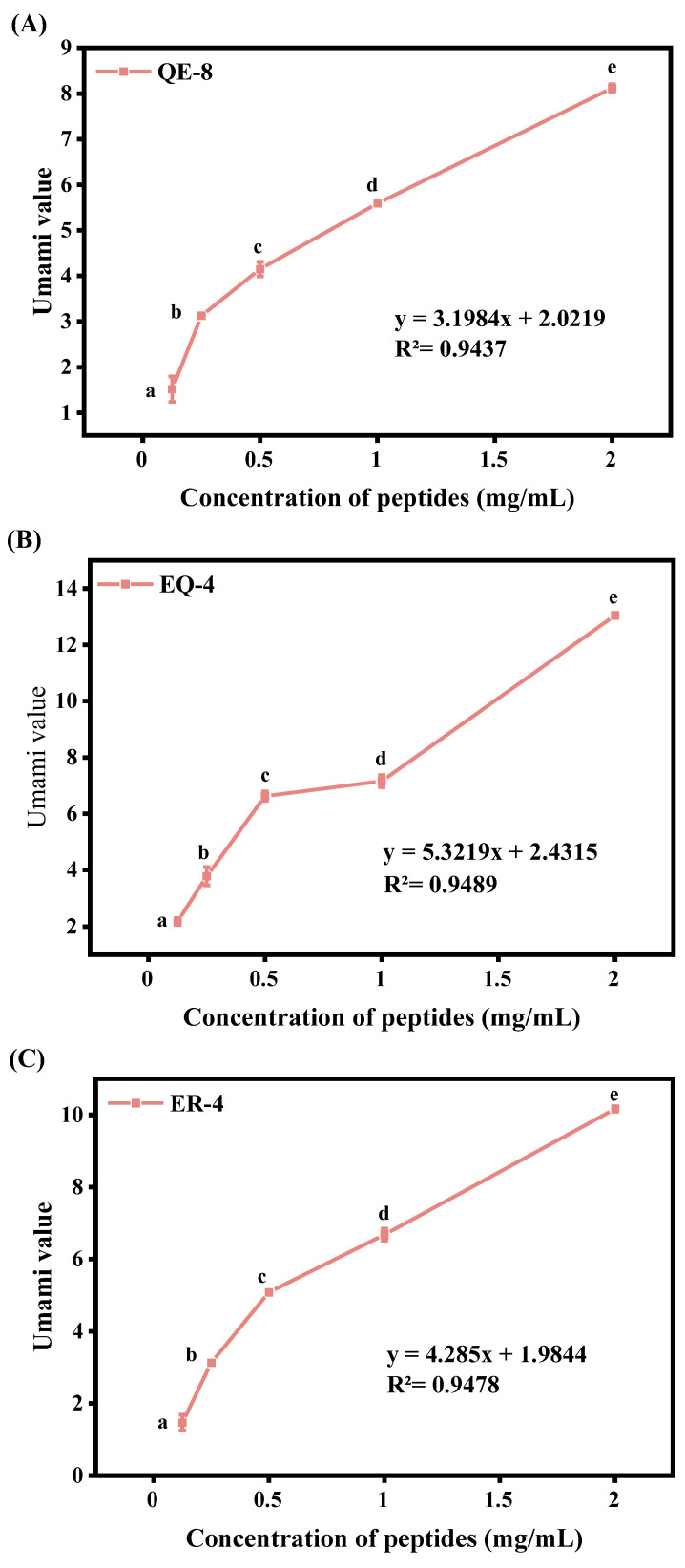

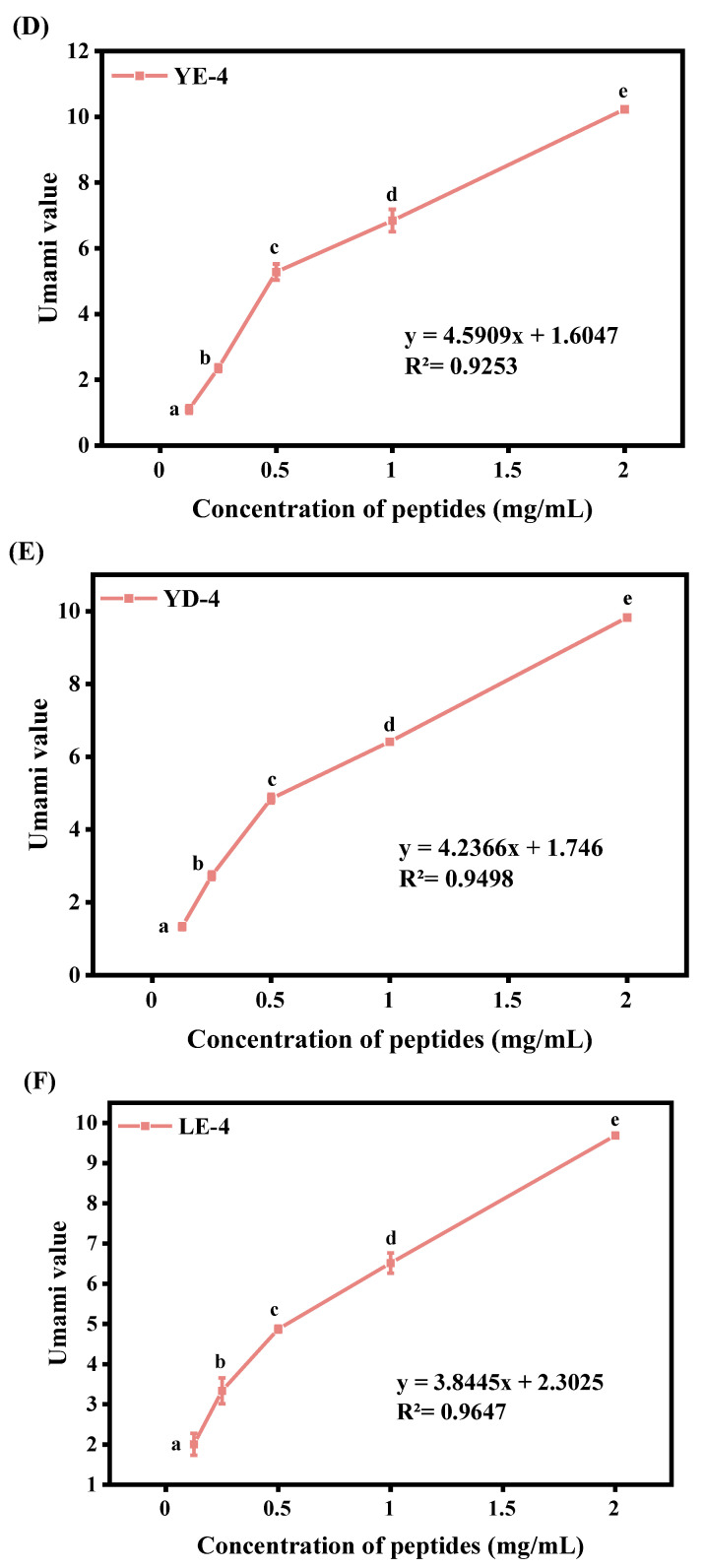
Quantity–effect relationship; (**A**): QE-8; (**B**): EQ-4; (**C**): ER-4; (**D**): YE-4; (**E**): YD-4; (**F**): LE-4. Different letters indicate significant differences between the data.

All six umami peptides exhibited umami activity (Figure 1A–F), with the intensity of this activity increasing in proportion to the concentration. The R2 value ranged from 0.9253 to 0.9647 within the concentration range of 0.125 mg/mL to 2 mg/mL, indicating that the umami intensity of the six peptides and the concentration exhibited a satisfactory linear relationship. In general, peptide EQ-4 exhibited the highest umami value in relation to the umami value of interest, followed by YE-4 and ER-4. According to prior research, the physicochemical properties and flavour profiles of peptides are frequently shaped by their amino acid composition [4]. It has been established through documented evidence that peptides comprising a continuous umami amino acid structure (e.g., EE, DD, ED, and DE) have a distinctive umami taste [24], as exemplified by QQLPQFEE, EELR, and EEDQ. Furthermore, the C-terminal or N-terminal residues of umami peptide sequences typically contain E or D, respectively, which contributes to the umami flavour [25], and all six umami peptides possess these features. Umami amino acids are known to activate taste receptors, while acidic amino acids have been identified as important umami stimulants [26]. The highest proportion of these two amino acids was found in EEDQ, which may account for the high umami intensity exhibited by this peptide [4]. In contrast, peptides such as YTCE, YTTD, and EEDQ are composed exclusively of hydrophilic amino acids. Studies have demonstrated that more hydrophilic amino acids may increase organoleptic receptivity [27]. In the case of medium-length peptides (QQLPQFEE), factors such as spatial structure play an even more critical role in determining their flavour properties [27].

### 3.2. Application of Umami Peptides in Condiments

**Figure 2:**
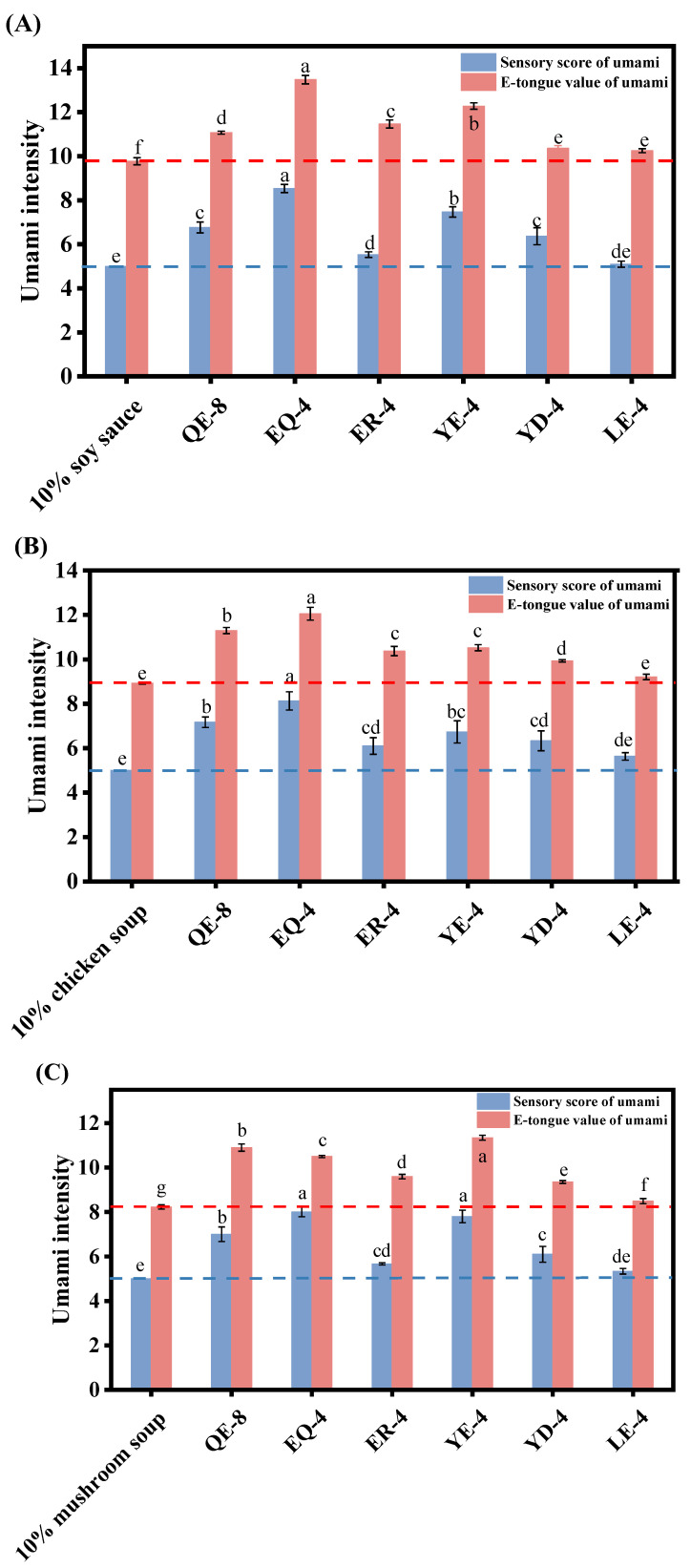

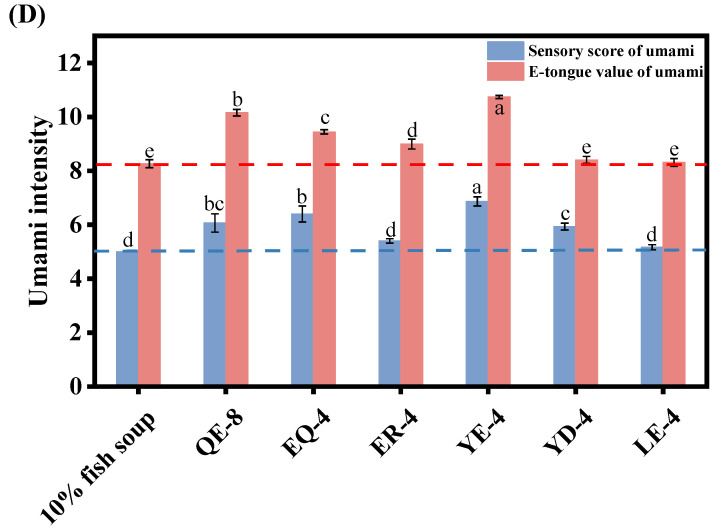
Application of umami peptides in (**A**) soy sauce; (**B**) chicken soup; (**C**) mushroom soup; (**D**) fish soup. Different letters indicate significant differences between the data. The red and blue dashed lines represent the umami intensity of the condiment solution without added umami peptides.

E-tongues employ taste bionic technology and are designed to emulate the functionality of human taste cells. The taste sensors of e-tongues, based on the principle of bilayer lipid membranes, are able to discern specific tastes and elicit dedicated responses. They produce taste signals by detecting changes in the membrane potential resulting from electrostatic or hydrophobic interactions between the taste substance and the artificial lipid membrane. The output of this response is not the number of taste-presenting substances, but a comprehensive assessment of their characteristics and intensity. Nevertheless, e-tongues are currently unable to provide a robust response signal for all the objects detected due to the constraints of the sensor array. Consequently, a comprehensive and objective evaluation of the effects of taste based on e-tongue results remains inadequate, and the process of sensory evaluation is prone to influence from the heterogeneity among individuals and changes in the sensory environment. Therefore, a combination of an e-tongue and sensory evaluation represents a promising approach for analysing the impact of umami with greater reliability. Furthermore, a substantial number of amino acid or peptide derivatives present in foodstuff, irrespective of their intrinsic flavour, can also manifest distinctive flavour properties when combined with other taste substances. Derivatives can play a significant role in enriching the fundamental taste of foods, although their concentrations in food matrices are typically below their corresponding organoleptic thresholds [28]. The addition of inorganic ions, such as sodium and chloride, has been shown to enhance the flavour of taste peptides in food due to synergistic effects [26]. Therefore, further investigation is required to elucidate the synergistic taste and enhancement effects of peptides in real food systems. Soy sauce, chicken soup, mushroom soup, and chicken soup created conditions for us to experiment in due to their abundance of taste substances. The umami enhancement effect of peptides on commercially available condiments is shown in Figure 2A–D. The six umami peptides had positive effects on the umami of common commercially available condiments (e-tongue), and the peptides with the strongest effects on the umami of the different condiments were as follows: EQ-4 in soy sauce and chicken soup (EQ-4), and YE-4 in mushroom soup and fish soup. These favourable outcomes may be attributed to the intricate interactions with other taste substances present in condiments. Meanwhile, the sensory evaluation indicated that QE-8, YE-4, and EQ-4 exhibited a superior umami enhancement effect on the seasoning solution in comparison to the other peptides. This finding aligns with a previous publication using sensory evaluations, namely in aqueous solutions, that showed that these three peptides exhibited better umami properties [4]. However, the results of the umami improvement in the condiments were not exactly the same in the e-tongue and sensory evaluations; this discrepancy could be attributed to the inherent differences in the response of human taste buds and electronic tongue biosensors in taste analyses.

### 3.3. Analysis of MD Simulation Results

**Figure 3:**
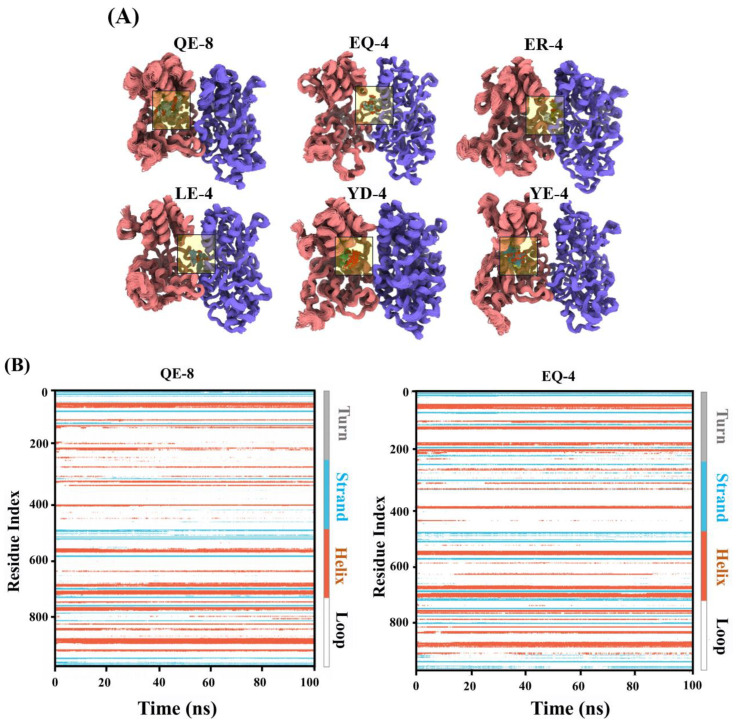

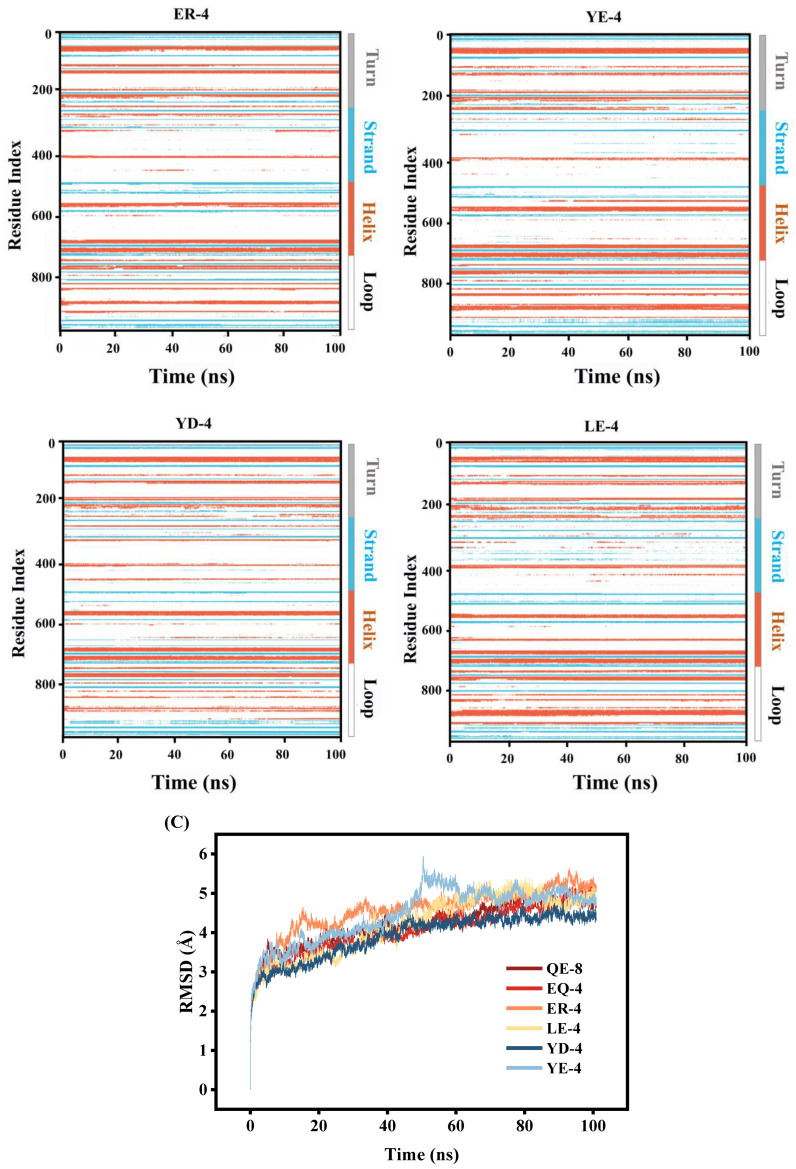

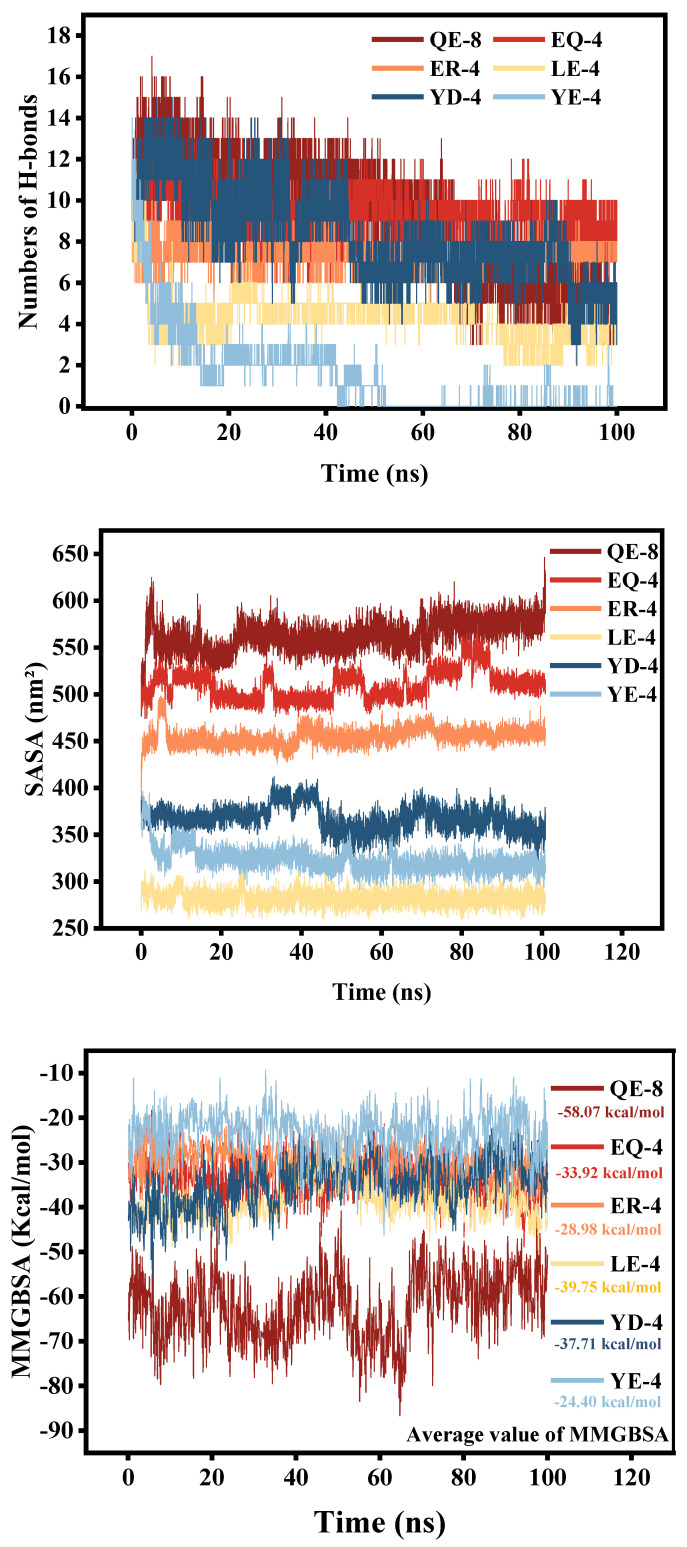

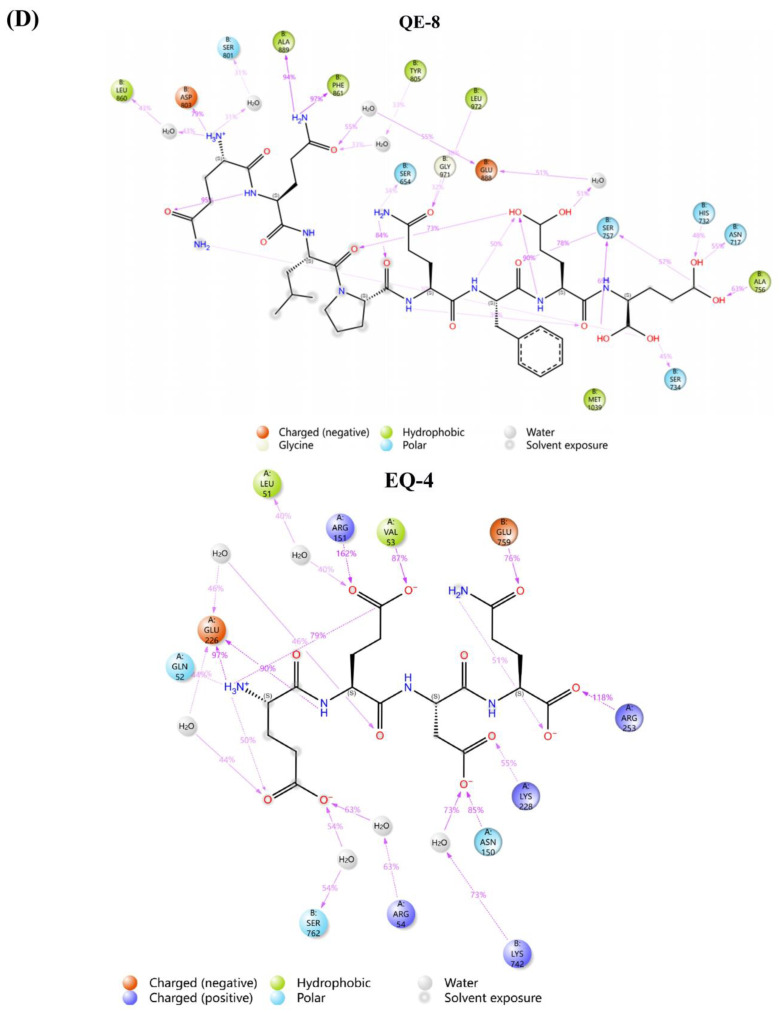

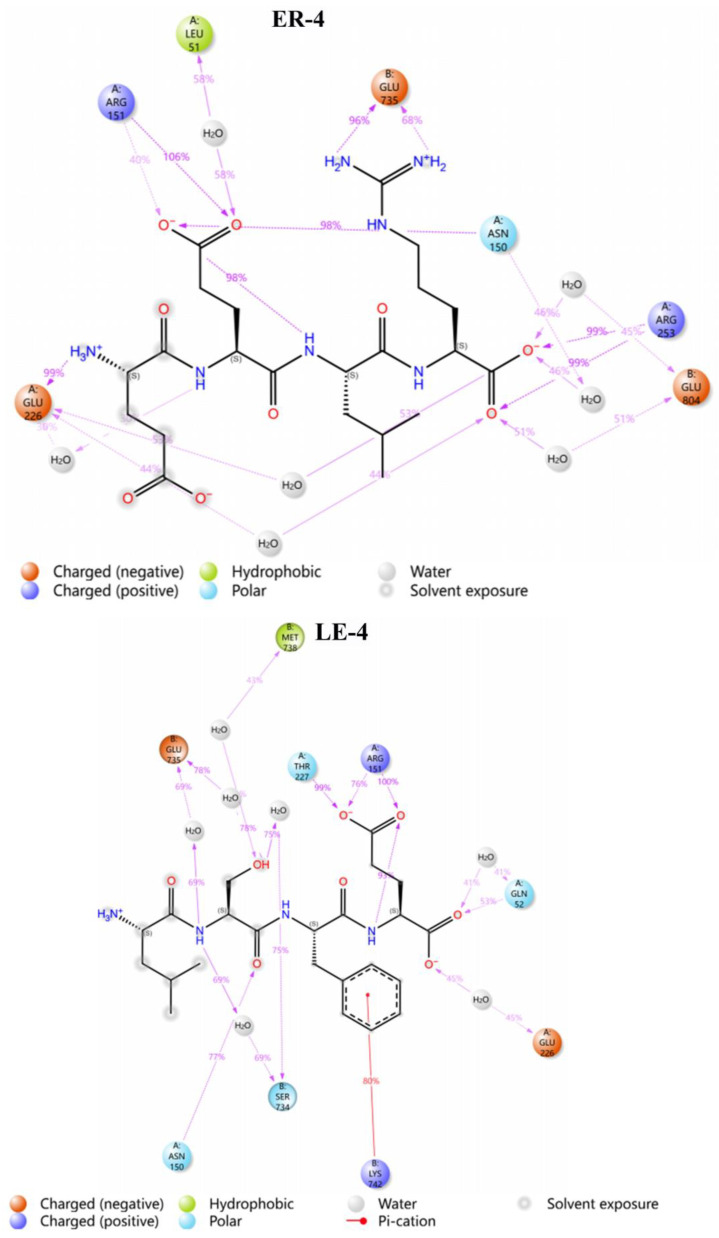

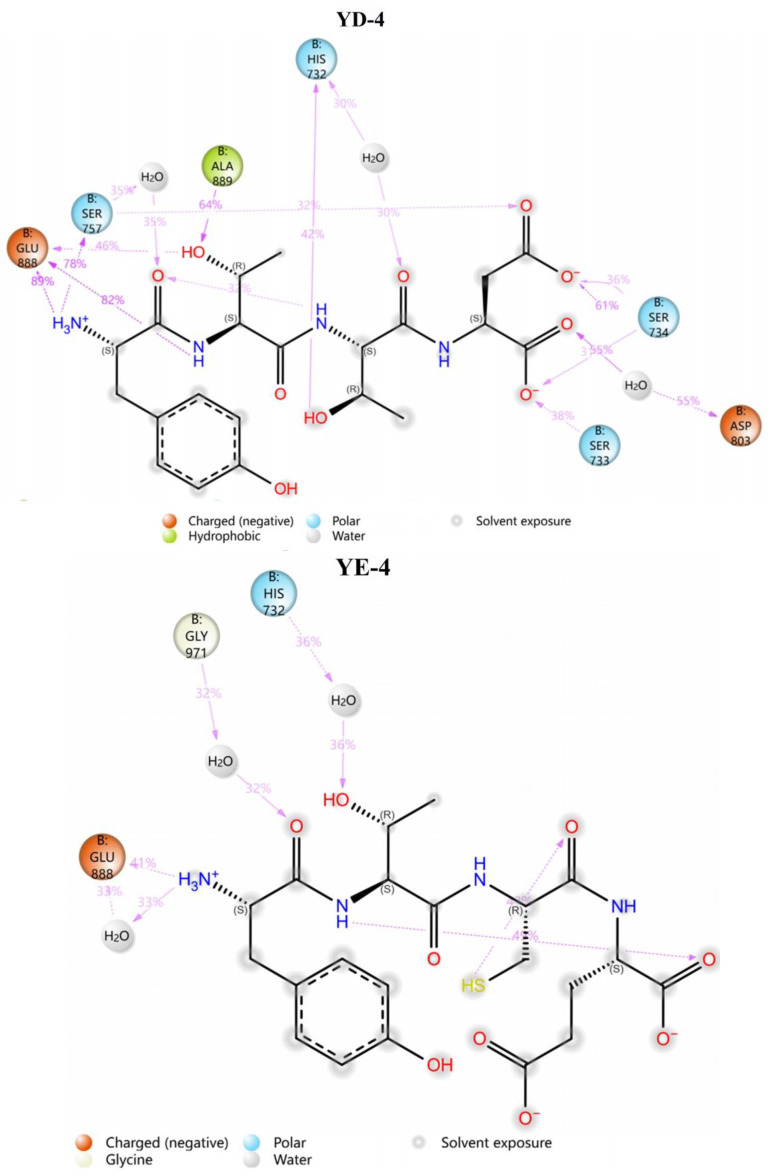

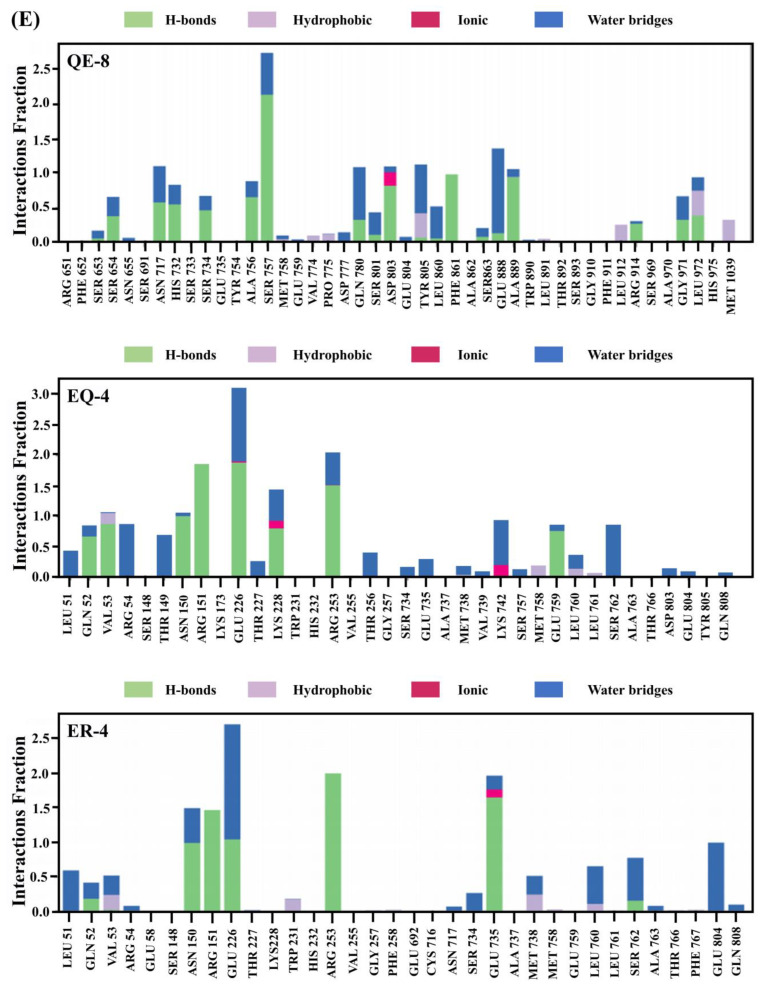

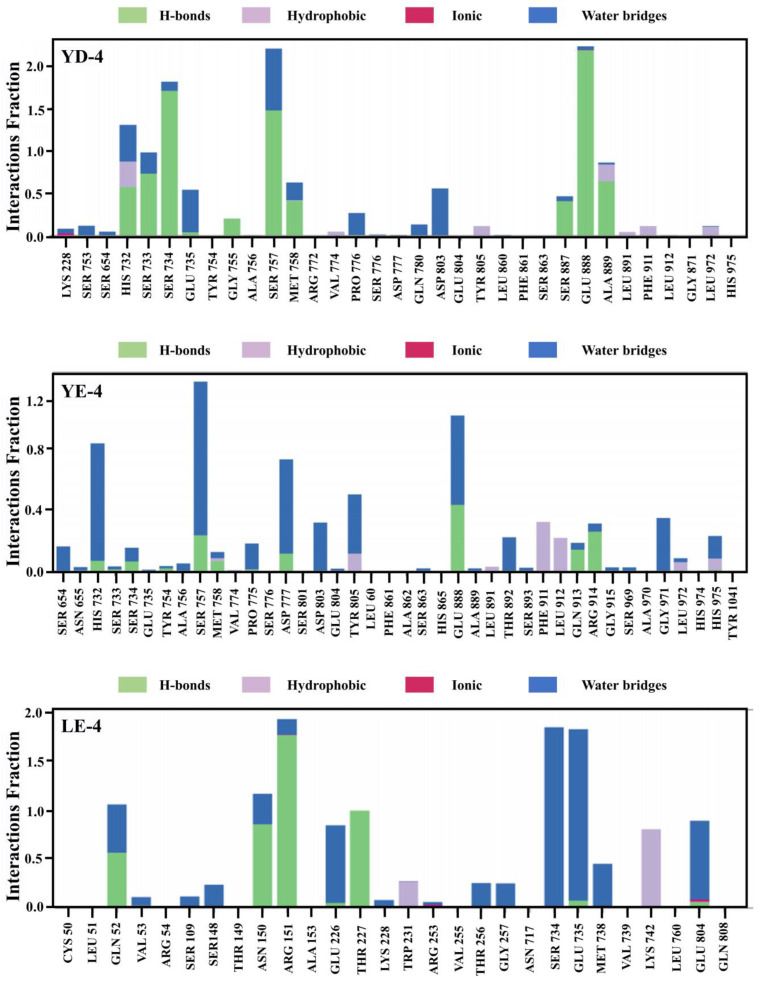
The results of the molecular dynamics simulations. (**A**) One hundred conformational overlays per 1 ns in 100 ns. (**B**) Plot of changes in secondary structure of proteins in 100 ns; different colours represent different protein secondary structure elements and fluctuations in the residue index represent the changes in protein secondary structure. (**C**) The results of the MD interactions during 100 ns simulations. (**D**) Two-dimensional schematic representation of the binding pattern of the peptides during the MD simulations with T1R1/T1R3 receptors. (**E**) Interaction forces during the MD simulations of the peptides and T1R1/T1R3 receptors.

Molecular dynamics simulations are a common tool for studying ligand–receptor interactions, allowing for the identification of docking targets based on affinity characteristics and the investigation of the dynamics between peptides and umami receptors [29]. Figure 3A illustrates the docking positions of the umami peptides and T1R1/T1R3 over a 100 ns period. The superposition of the 100 conformations of the six umami peptides revealed a high degree of consistency, indicating that the conformations exhibited a considerable degree of stability throughout the simulation. This suggests that the peptides were able to stably bind to the active sites of the proteins. Figure 3B illustrates the secondary structure alterations of the peptides and receptors throughout the 100 ns binding period. Different colours represent different protein secondary structure elements, and fluctuations in the residue index represent the changes in the proteins’ secondary structure. The residue index of strands, helices, and loops at each position remained basically unchanged, indicating that the overall secondary structure of the proteins was essentially unaltered during molecular dynamics simulation process. This suggests that the composite conformation of the T1R1/T1R3 receptors and the peptides could be maintained during the interaction.

The results of the RMSD, number of H bonds, SASA, and MMGBSA are presented in Figure 3C. RMSD can assess the stability of protein–ligand complexes, and a reduction in the fluctuations of the RMSD curves indicates an increase in the stability of the complexes. The RMSD curves indicate that the peptides exhibited significant fluctuations during the initial 10 ns of the simulation, which may be attributed to the extensive flexible jitter between the peptides and the receptors [29]. The RMSD values of the proteins and peptides appeared to stabilise after 20 ns, indicating that the protein–peptide complex reached a position of relative stability. To explore the nature of the hydrogen bonding in the conjugation region of the peptide–T1R1/T1R3 complex, the number of hydrogen bonds produced in the stabilised structure was calculated in this study. The number of hydrogen bonds exhibited fluctuations throughout the simulation process, with a range of 0–14. In addition, the number of hydrogen bonds in the QE-8, EQ-4, ER-4, and YD-4 complexes stabilised at or above five during the process, indicating that the complexes formed were tight and robust. In general, QE-8 exhibited the highest number of hydrogen bonds, which may be attributed to the relatively long peptide chain length, which is more likely to engage in hydrogen-bonding interactions with the receptors. Conversely, the number of hydrogen bonds in LE-4 and YE-4 was low or even absent, which may result in a reduction in the stability of the complexes formed between them and the receptors [30]. Furthermore, the variations in the structure and properties of the T1R1/T1R3 proteins were analysed by molecular dynamics (MD) simulations. The water molecules surrounding the receptors exhibited robust interactions with the amino acids of the umami receptors throughout the kinetic process, resulting in fluctuations in the SASA value. Satisfyingly, the SASA values of the six polypeptides demonstrate consistent stability, indicating that the interaction between water molecules and the umami receptor proteins was mostly stable. In particular, the SASA value of LE-4 was relatively small, whereas that of QE-8 was the largest. This may be due to the more complex structure of QE-8, which resulted in its binding near the active pocket and exposure on the surface of proteins. Consequently, the SASA value of this complex was relatively large [31]. The aforementioned indicators serve as pivotal determinants in the stability of the binding process between umami peptides and T1R1/T1R3.

MMGBSA is a measure of the binding energy between peptides and their receptor. A higher absolute value of the interaction energy indicates a stronger interaction. The mean MMGBSA of the six peptides ranged from −24.40 to −58.07 kcal/mol, and all the peptides were observed to stably bind to the umami receptors T1R1/T1R3. The strongest binding ability was demonstrated by QE-8 (−58.07 kcal/mol), indicating that its binding to the receptors was the most stable, which may be attributed to its complex structure that can form more interaction forces with the receptor, thus stabilising the complex conformation [29]. The free energy of MMGBSA binding between the proteins and peptides was decomposed to each specific amino acid of the proteins (A-chain: T1R1; B-chain: T1R3). The amino acids that are important for peptide binding are shown in Table 1 (the top 10 amino acid residues of binding energy were selected for display). The higher the absolute value of the energy, the higher the contribution of the amino acid residue to the stabilisation of the conformation, and amino acids with a high value are potential key binding sites. EQ-4, ER-4, and LE-4 bound to amino acid residues on the A and B chains. However, QE-8, YD-4, and YE-4 predominantly interacted with the amino acid residues on the B chain, indicating that these three peptides tend to interact with the T1R3 receptor. TRY805 (−6.45 kcal/mol), ARG151 (−5.69 kcal/mol), ARG151 (−4.44 kcal/mol), ARG151 (−6.40 kcal/mol), GLU888 (−6.58 kcal/mol), and HIS732 (−3.48 kcal/mol) are the key amino acid residues in QE-8, EQ-4, ER-4, LE-4, YD-4, and YE-4, respectively. These amino acid residues may play an irreplaceable role in stabilising the conformation of the peptide and receptor complexes, while residue ARG151 in the A-chain is likely to be the key site for recognising the binding of umami peptides to the T1R1/T1R3 receptors.

Following the statistical analysis of the interaction patterns observed in the 50–100 ns kinetic simulation trajectories, the occupancy of each interaction formation (i.e., the ratio of the number of frames in which an interaction was formed to the total number of frames) was obtained, which is illustrated in Figure 3D. The polypeptides primarily relied on the side-chain carboxyl groups and the amide backbones of the compounds to interact with the surrounding amino acids, either directly or indirectly through water molecules. These interactions included charged, hydrophobic, water, glycine, polar, solvent hydrophobic, water, glycine, polar, solvent exposure, and Pi-cation interactions, which together stabilised the binding of the peptide to the T1R1/T1R3 receptors. A detailed examination of the interaction patterns within the stabilisation interval of the kinetic trajectory revealed that specific amino acids play a pivotal role in facilitating T1R1/T1R3-receptor binding (interaction frequency > 1), as illustrated in Figure 3E. QE-8 had the largest number of major residues including ASN 717, SER 757, GLN 780, ASP 803, TYR 805, GLU 888, and ALA 889. VAL 53, ASN 150, ARG 151, GLU 226, LYS 228, and ARG 253 were important residues in EQ-4. The major residues in ER-4 were ASN 150, ARG 151, GLU 226, ARG 253, GLU 735, and GLU 804. The key residues of LE-4 were GLN 52, ASN 150, ARG 151, SER 734, and GLU 735, whereas HIS 732, SER 734, SER 757, and GLU 888 were mainly involved in the stabilisation of the YD-4 conformation. In addition, YE-4 only had two important residues: SER 757 and GLU 888. These residues help us to understand how umami peptides interact with T1R1/T1R3 receptors. They are now a novel reference site for studying umami peptides. Furthermore, the molecular dynamics process found several types of interactions between the peptides and receptors, including hydrogen bonds, hydrophobic interactions, ionic interactions, and water bridges. These interactions (including hydrogen bonds and hydrophobic interactions) enhanced the overall binding stability and affinity of the conformation of the peptides and umami receptors, which is in agreement with the findings of previous studies [26]. Although a variety of interactions have been reported during molecular binding, there is no method to accurately identify the binding sites and interactive forces involved. In contrast to the simplicity of amino acid molecules, when investigating the binding mechanism of umami peptides to their receptors, more influencing factors, such as the molecular properties and chain flexibility of the peptide, should be taken into account.

### 3.4. Antioxidant Activity Analysis

**Figure 4:**
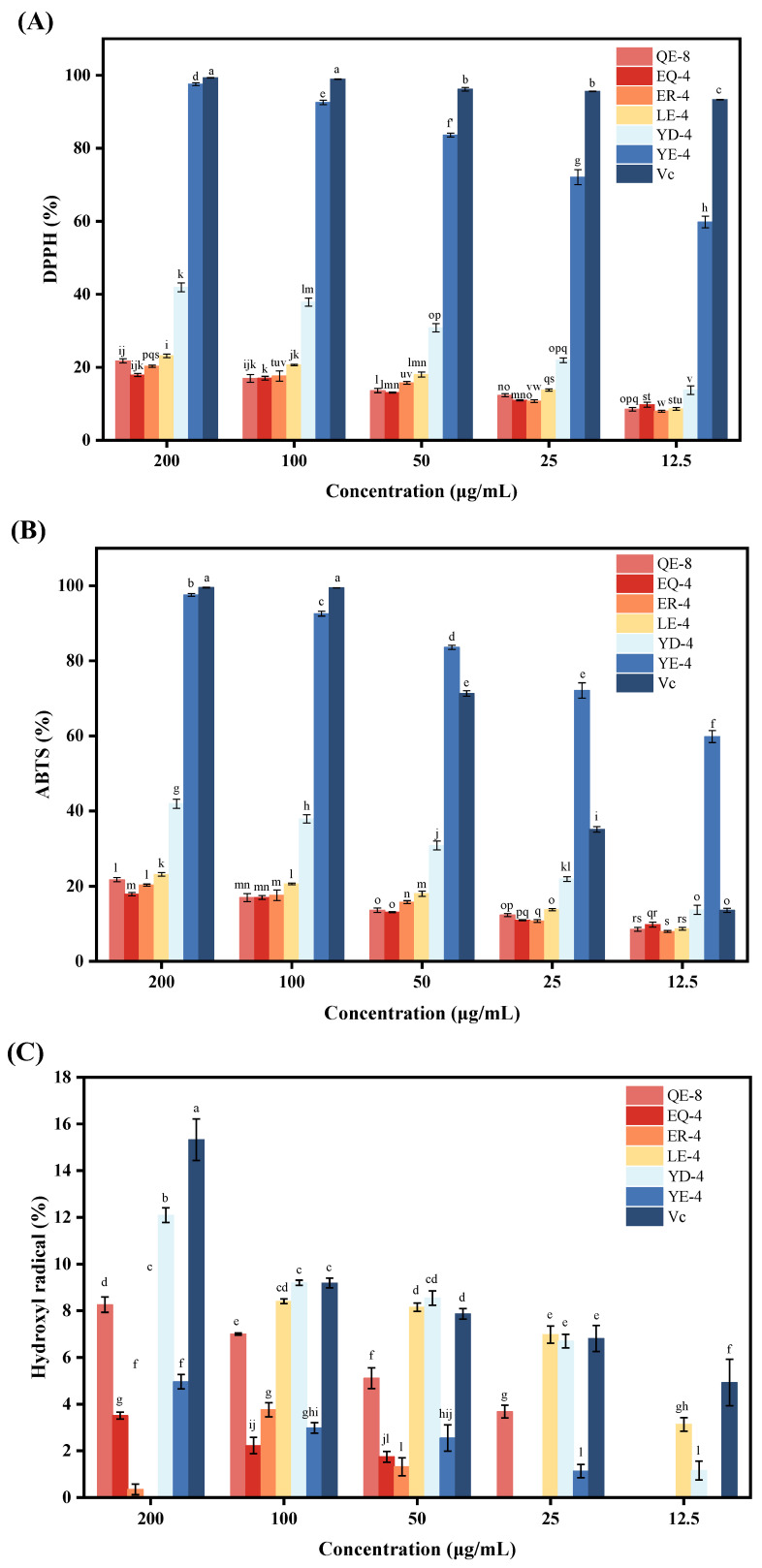

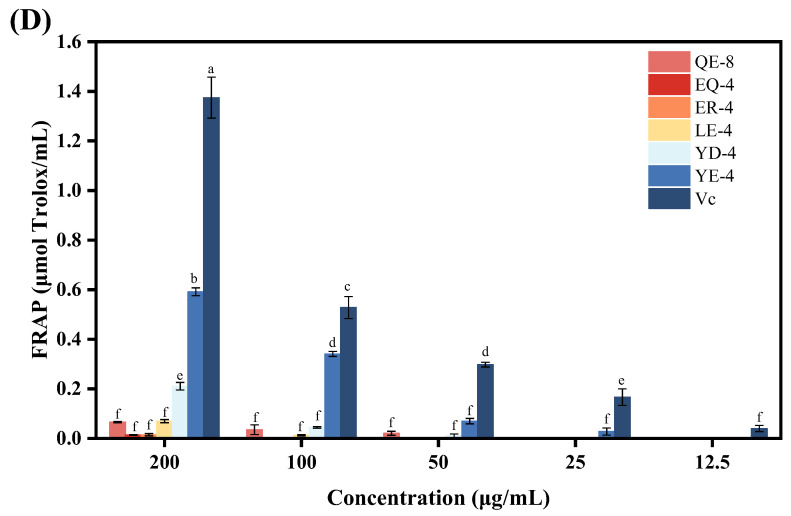
The results of antioxidant activity assays; (**A**) DPPH; (**B**) ABTS; (**C**) hydroxyl radicals; (**D**) FRAP. Different letters indicate significant differences between the data.

The antioxidant activity of the six umami peptides was evaluated using the DPPH, ABTS, hydroxyl radical-scavenging, and FRAP assays. Additionally, the dose–response relationship was investigated. However, the applicability of these different assays varies. For instance, the DPPH method is considered an appropriate approach for the evaluation of antioxidant activity in hydrophobic substances, whereas the ABTS method is deemed more suitable for assessments under hydrophilic and lipophilic antioxidant conditions. In contrast, the hydroxyl radical and FRAP methods primarily act on metal ions to assess antioxidant capacity [7]. As illustrated in Figure 4A–D, all six umami peptides demonstrated varying degrees of antioxidant activity, and their antioxidant activity weakened with decreasing concentration in the range of 200 μg/mL to 12.5 μg/mL. The antioxidant activity of peptides is mainly contingent upon their compositional make-up and the nature of the amino acids. The exposed structure of hydrophobic amino acid residues plays a pivotal role in determining their antioxidant activity. QE-8, LE-4, and ER-4 contain hydrophobic amino acids, and therefore it is not surprising that they possess antioxidant activity [7]. However, an elevated concentration of hydrophobic amino acids in peptides affects their solubility, which may reduce their capacity to scavenge radicals. In comparison, YE-4, YD-4, and EQ-4 are all composed of hydrophilic amino acids, which are conducive to the production of umami flavour. Additionally, it has been reported that the participating antioxidant amino acids are aspartic acid, glutamic acid, proline, and arginine [32]. The six peptides in the current study from WGs contain either aspartic acid or glutamic acid, which are amino acids that contribute to umami flavour. The six peptides from WGHs and fermented WGHs contain either aspartic acid or glutamatergic umami amino acids, which may be the reason why they all exhibit antioxidant potential. Among the peptides, YE-4 demonstrated the most promising antioxidant activity, exhibiting the highest DPPH- (Figure 4A) and ABTS-scavenging (Figure 4B) rates and a notable iron-reducing capacity (Figure 4D). Notably, the ABTS-scavenging rate of YE-4 surpassed that of vitamin C in the concentration range of 50 µg/mL to 12.5 µg/mL. Additionally, YD-4 exhibited an exceptional hydroxyl radical-scavenging rate (Figure 4C). Taken together, the antioxidant capacities of the six peptides were ranked as follows: YE-4 > YD-4 > QE-8 > LE-4 > ER-4 > EQ-4. While the antioxidant efficacy of these peptides may not reach the level of the positive control VC in the majority of cases, they nevertheless demonstrated a notable antioxidant capacity. Consequently, these peptides may have promise in applications in the field of antioxidants.

### 3.5. Network Pharmacology Analysis

#### 3.5.1. Prediction of Umami Peptides and Disease Targets

**Figure 5:**
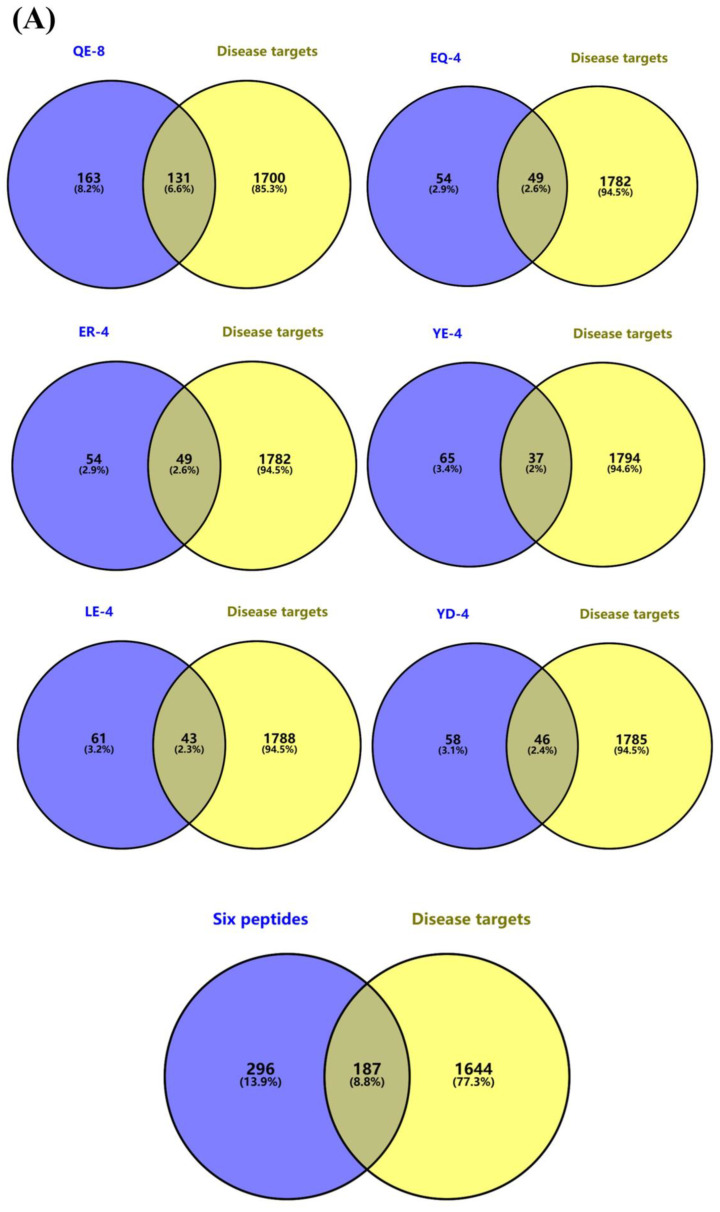

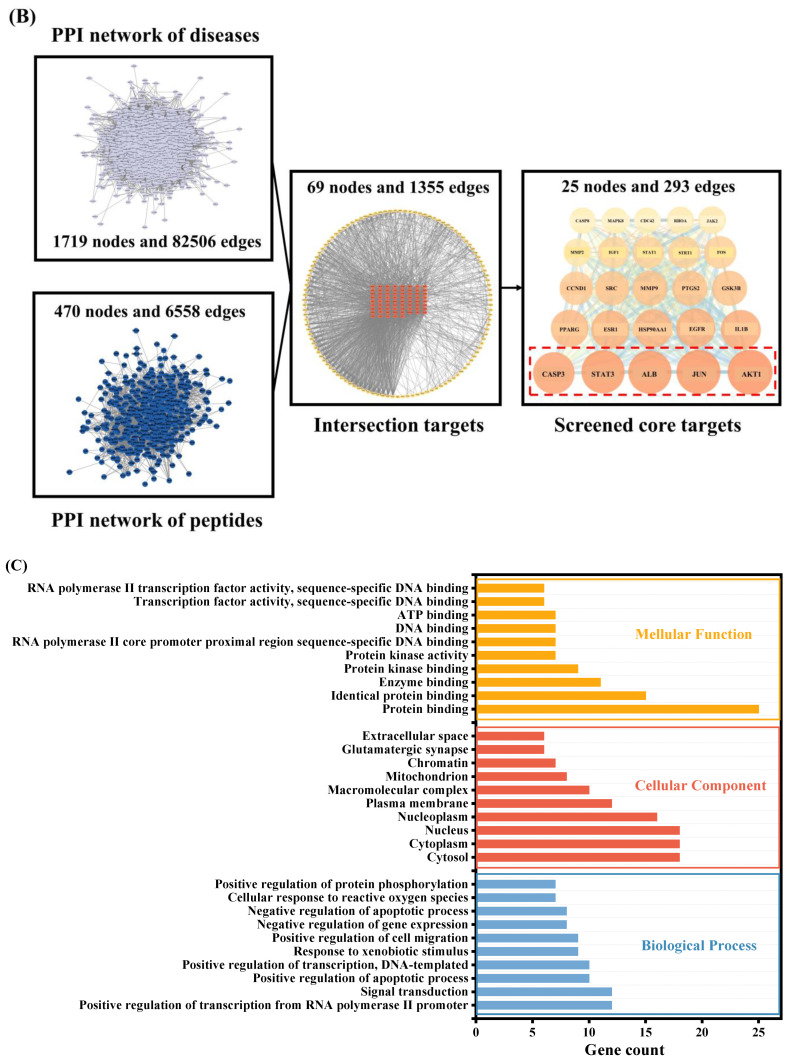

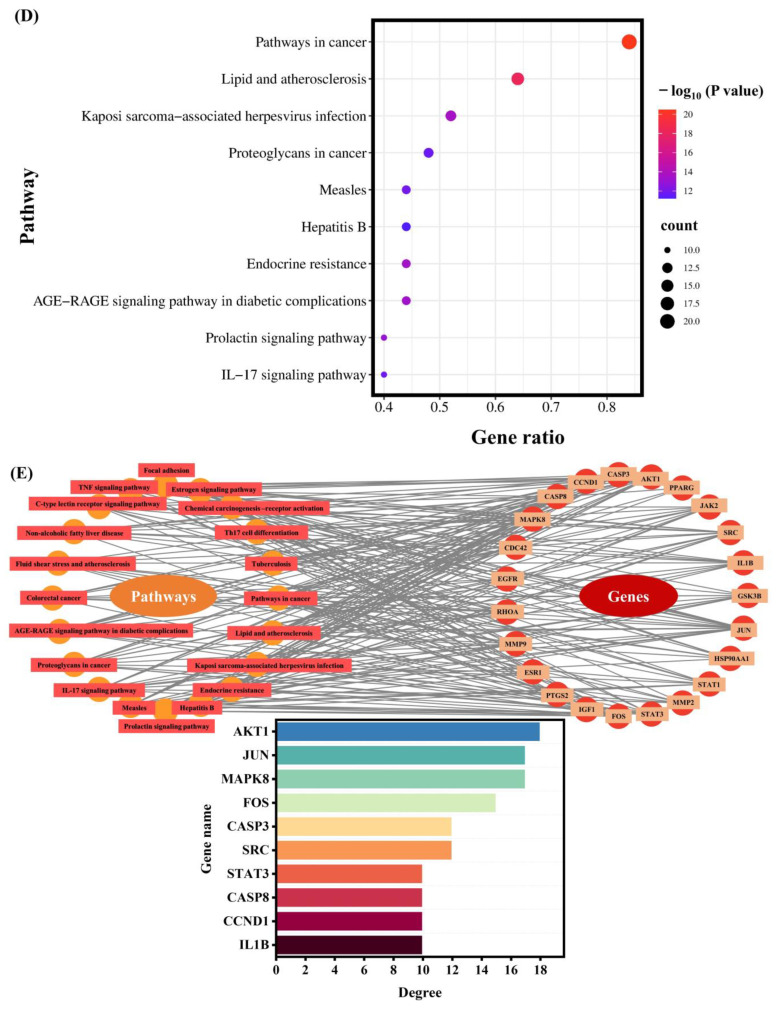
The results of the network pharmacology analysis; (**A**) Venn diagrams of peptide versus disease target genes; (**B**) screening process for key nodes of core targets and peptides in disease; (**C**) GO enrichment analysis results; (**D**) KEGG pathway analysis results; (**E**) the network of the key targeted pathways.

The OMIM, DisGeNET, and GeneCards databases were employed to retrieve oxidation-associated targets, and the disease target networks for each peptide were constructed using Cytoscape 3.8.2. As illustrated in Table S1 (Supplementary Materials), the number of targets associated with QE-8, LE-4, ER-4, YE-4, YD-4, and EQ-4 were 295, 110, 108, 105, 107, and 106, respectively. The relatively short peptides retrieved a similar number of targets. However, QE-8 was found to have a greater number of relevant targets associated with oxidative diseases, which can be ascribed to its more complex structure of long peptide fragments. It is noteworthy that the Venn diagram analysis showed common targets between the peptides and oxidative diseases (Figure 5A). The number of intersections between the six umami peptides and disease targets ranged from 37 to 131. After deleting the duplicates in the peptide targets, a total of 296 gene targets were obtained. Ultimately, 187 (8.8%) umami peptide–antioxidant intersections were obtained, which may have a role in these diseases.

To elucidate the significance of the six umami peptides in combating oxidative diseases, two target networks were built using the Cytospace 3.8.2 software. The peptides’ targets of action and the antioxidant targets were loaded into the Cytospace 3.8.2 software from the STRING database. As illustrated in Figure 5B, the peptide target network comprised 470 nodes and 6558 connections, while the antioxidant disease target network encompassed 1719 nodes and 82,506 connections. The combination of the two target networks allowed for the identification of associations between the peptides’ predicted disease targets and disease-related targets. This resulted in the generation of an overlapping network between the peptides’ targets of action and disease targets, comprising 69 nodes and 1355 connections. Subsequently, the core targets, the top 25 ranked targets, were subjected to further investigation. These targets are of pivotal importance in a wide range of interrelated responses that are closely linked to oxidative diseases. For example, AKT1 and STAT3 have been demonstrated to be part of the signalling pathway involved in the growth and metastasis of osteosarcoma [33]. JUN and CASP3 have also been shown to be one of the key factors causing Burkitt lymphoma. These findings offer a penetrating view into potential disease treatments using umami peptides [34].

#### 3.5.2. GO and KEGG Enrichment Analyses

In order to gain further insight into the mechanism of the antioxidant activity of the umami peptides, the GO functionss and KEGG pathway enrichment of the 25 core target genes were analysed using the DAVID database. Figure 5C shows the top 10 GO entries associated with biological processes (BPs), cellular components (CCs), and molecular functions (MFs). For example, the BPs mainly included protein binding, identical protein binding, enzyme binding, protein kinase binding, and protein kinase activity. Regarding CCs, notable entries included cytosol, cytoplasm, nucleus, nucleoplasm, and plasma membrane. In terms of MFs, the enriched terms mainly included positive regulation of transcription from RNA polymerase II promoters, signal transduction, positive regulation of apoptotic processes, and positive regulation of DNA-templated transcription. These GO enrichment results are similar to those reported in an antioxidant mechanism analysis of peptides from walnut and citrus herbs [22,35]. These results demonstrate that umami peptides might alleviate oxidative stress-induced damage by regulating biological processes across diverse cellular compartments and functions.

Furthermore, in order to elucidate the pharmacological mechanisms of peptide therapeutics in oxidative diseases in a systematic manner, a KEGG analysis was employed to pinpoint the potential signalling pathways involving the targets (Figure 5D). The results indicate that umami peptides may be involved in numerous pathways associated with oxidative diseases (the top 10 entries are shown), including cancer, lipid and atherosclerosis, Kaposi sarcoma-associated herpesvirus infection, proteoglycans in cancer, measles, hepatitis B, endocrine resistance, AGE-RAGE signalling in diabetic complications, prolactin signalling, and IL-17 signalling pathways. The KEGG pathway analysis revealed that “pathways in cancer” and “lipid and atherosclerosis” pathways were the most prominent in terms of gene enrichment. This finding is consistent with previous network pharmacology studies on antioxidants [22]. It is well established that the development of cancer and cardiovascular-related diseases is closely associated with oxidative stress [36,37]. The aforementioned pathways may provide a theoretical foundation for the use of these six umami peptides in the treatment of related diseases.

A target pathway network map was developed based on the key targets of the umami peptides and KEGG pathways, as well as their interactions. The core targets were then ranked on the basis of the degree values in order to facilitate further analysis. The resulting diagram is shown in Figure 5E. AKT1, JUN, MAPK8, FOS, and CASP3 were implicated in a multitude of pathways and may represent the core targets of peptide therapy for diseases. Additionally, the genes AKT1, JUN, and CASP3 were identified as among the top five targets in Figure 5B. Consequently, these three disease-associated genes were chosen for subsequent molecular docking studies to elucidate the prospective therapeutic roles that may be exerted by umami peptides.

### 3.6. Molecular Docking Analysis

**Figure 6:**
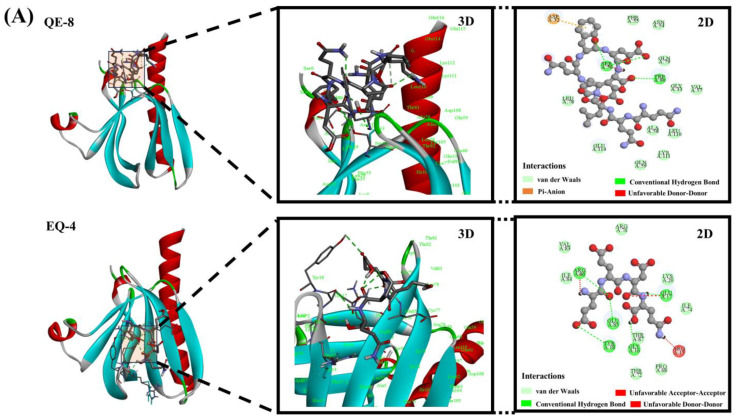

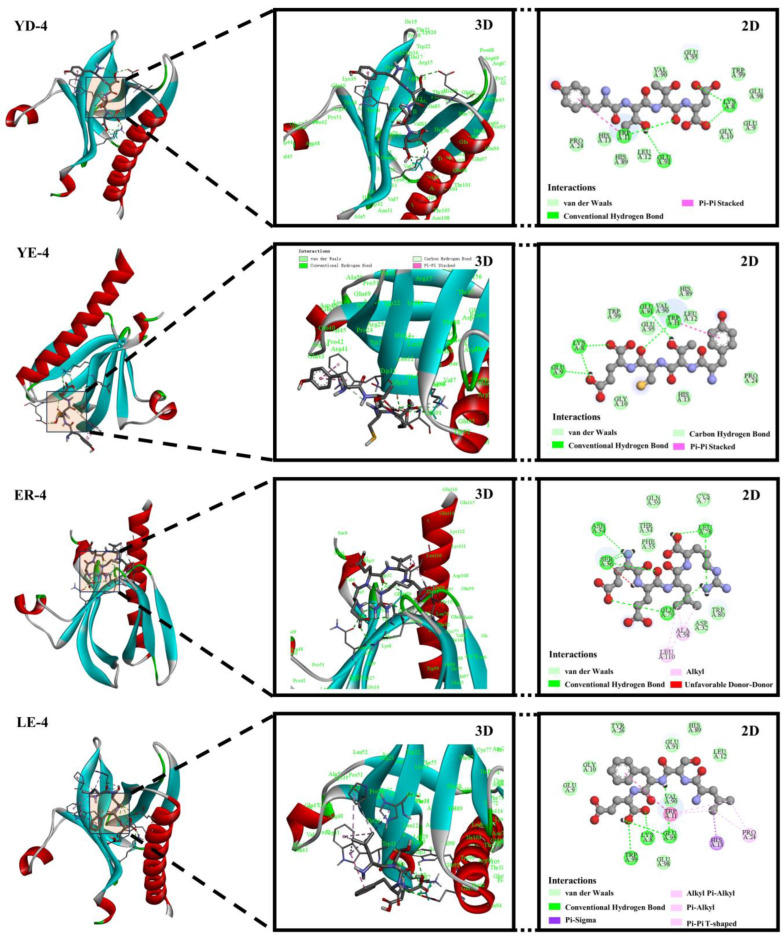

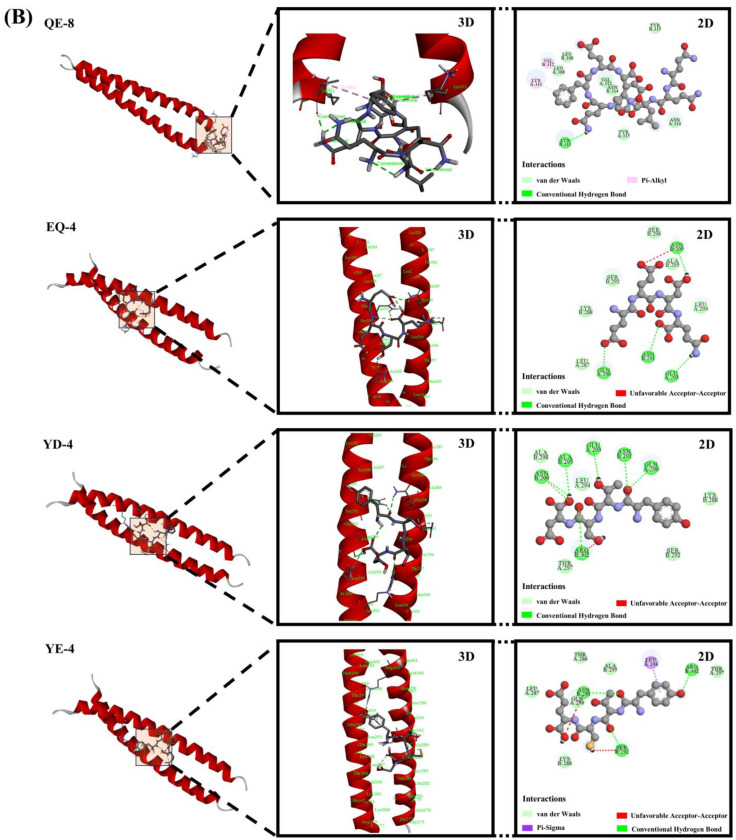

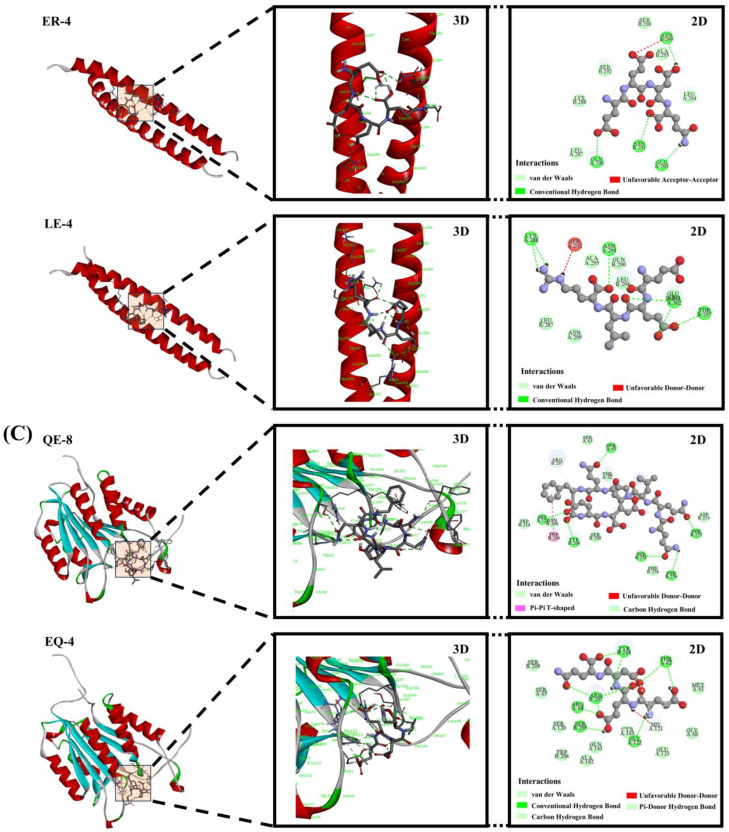

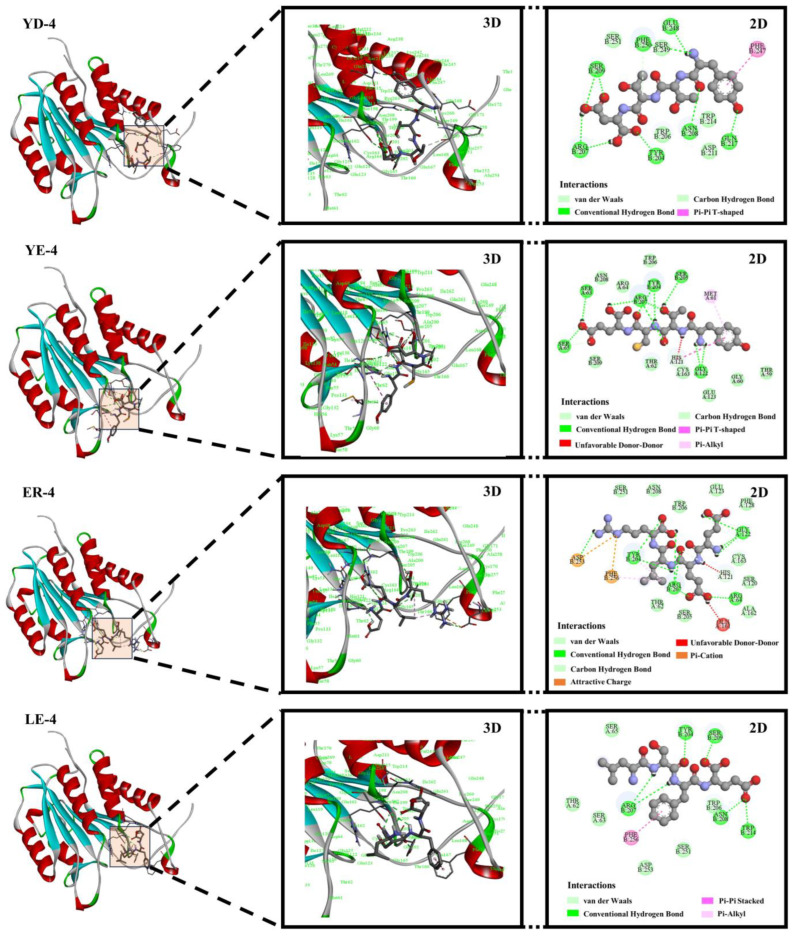
Molecular docking results between peptides and targets; (**A**) docking results for peptides and AKT1; (**B**) docking results for JUN; (**C**) docking results for CASP3.

The results of the network pharmacology screening identified RAC-alpha serine/threonine protein kinase 1 (AKT1), jun proto-oncogene (JUN), and caspase-3 (CASP3) as potential molecular targets for further study. These core disease targets (AKT1, JUN, and CASP3) have been demonstrated to play crucial roles in the pathogenesis of diabetes, cancer, and heart disease [38]. The molecular docking results are illustrated in Figure 6A–C, which show that the six peptides can bind to different target receptors through different active amino acid residues. In docking with AKT1, hydrogen-bonding interactions (van der Waals interactions, conventional hydrogen bonds, and carbon–hydrogen bonds), hydrophobic interactions (alkyl, pi-pi T-shaped, and pi-alkyl), and electrostatic interactions (pi-anion) were identified as the main forces stabilising the binding conformation. In addition, hydrogen-bonding interactions (van der Waals, conventional hydrogen bonding, carbon–hydrogen bonding, pi-donor hydrogen bonding), hydrophobic interactions (pi-pi stacked and pi-alkyl), and electrostatic interactions (attraction of charge and pi-cation) were the types of forces involved in the docking of the umami peptides to CASP3. In contrast, only hydrogen-bonding interactions (van der Waals and conventional hydrogen bonds) and hydrophobic interactions (pi-alkyl and pi-sigma) were implicated in the interaction between the peptides and JUN. Three principal interactions that have been demonstrated to stabilise receptor–ligand complexes are hydrophobic interactions, electrostatic interactions, and hydrogen bonding [39]. These findings are in accordance with the results of the present experiment. Furthermore, conventional hydrogen bond and van der Waals interactions appeared most frequently during the docking of umami peptides with the three disease targets, and it can be speculated that they have an important contribution in maintaining the binding stability between the peptides and the targets.

In an effort to estimate the affinity between peptides and their targets, the binding energy is typically modelled. It provides an indication of the possibility of binding between a receptor and ligand. A lower binding energy corresponds to a higher affinity and a more stable conformation [40]. The docking affinities of the umami peptides and their respective targets are presented in Table 2. The average affinity ranged from −5.97 to −6.57 kcal/mol, indicating a favourable docking activity between the peptides and their targets. The six peptides demonstrated the most robust binding affinity to AKT1, suggesting that these umami peptides may possess the most promising therapeutic potential for targeting AKT1. Conversely, YD-4 and YE-4 exhibited the strongest average binding affinity to the three targets. Moreover, YE-4 exhibited exceptional performance in in vitro antioxidant experiments, indicating that it may represent a promising high-quality antioxidant umami peptide.

## 4. Conclusions

In this research, an e-tongue was used to ascertain the umami intensity of umami peptides (QQLPQFEE, LSFE, EELR, YTCE, YTTD, and EEDQ) derived from WGHs and fermented WGHs, which exhibited dose-dependent effects. Furthermore, we confirmed the umami improvement effect of the six umami peptides in soy sauce, chicken soup, mushroom soup, and fish soup, which are similar condiments. The conformational changes, stability, and interaction mechanisms of the umami peptides and umami T1R1/T1R3 receptor complex were investigated through 100 ns molecular dynamics (MD) simulations. The in vitro antioxidant experiments indicated that the six umami peptides exhibited antioxidant activity. Among these, YE-4 stood out for its excellent umami taste property and antioxidant activity. A target model was constructed based on network pharmacology to elucidate the complex interactions between the flavour peptides and disease targets. The results indicated that the antioxidant activity of the peptides may be dependent on the activation of multiple signalling pathways. These KEGG pathways are implicated in cancer, lipid diseases and atherosclerosis, and other diseases. Molecular docking studies were conducted to complement the aforementioned analyses, which revealed strong binding effects between the peptides and AKT1, JUN, and CASP3, which are mainly mediated by hydrogen bonding. However, additional experiments are required to confirm whether the umami peptides can play a therapeutic role in oxidative diseases in organisms. In summary, our findings contribute to our comprehension of the mechanism of peptide umami generation and they underscore the prospective applications of peptides in taste sensation elevation and nutrition value enhancement in food formulations.

## Figures and Tables

**Table 1:** The binding energy of key amino acid residues.

QE-8		EQ-4		ER-4		LE-4		YD-4		YE-4	
Residue ID	Energy (kcal/mol)	Residue ID	Energy (kcal/mol)	Residue ID	Energy (kcal/mol)	Residue ID	Energy (kcal/mol)	Residue ID	Energy (kcal/mol)	Residue ID	Energy (kcal/mol)
B:805(TYR)	−6.45	A:151(ARG)	−5.69	A:151(ARG)	−4.44	A:151(ARG)	−6.40	B:888(GLU)	−6.58	B:732(HIS)	−3.48
B:757(SER)	−4.56	B:761(LEU)	−4.88	A:253(ARG)	−4.00	B:738(MET)	−4.15	B:889(ALA)	−3.50	B:912(LEU)	−2.04
B:861(PHE)	−2.56	B:758(MET)	−4.68	B:738(MET)	−3.42	A:150(ASN)	−4.14	B:756(ALA)	−2.85	B:972(LEU)	−1.80
B:972(LEU)	−2.54	A:228(LYS)	−3.55	A:53(VAL)	−2.43	A:226(GLH)	−2.84	B:733(SER)	−2.58	B:757(SER)	−1.48
B:971(GLY)	−2.43	A:54(ARG)	−2.98	B:735(GLU)	−2.14	A:227(THR)	−2.59	B:757(SER)	−2.51	B:911(PHE)	−1.23
B:970(ALA)	−2.34	B:760(LEU)	−2.79	A:150(ASN)	−1.99	B:742(LYS)	−2.20	B:732(HIS)	−2.21	B:891(LEU)	−1.08
B:862(ALA)	−2.11	A:253(ARG)	−2.75	B:758(MET)	−1.97	A:231(TRP)	−1.58	B:972(LEU)	−1.20	B:756(ALA)	−1.03
B:914(ARG)	−2.02	A:52(GLN)	−2.70	B:761(LEU)	−1.84	A:128(GLH)	−1.16	B:805(TYR)	−1.04	B:755(GLY)	−0.98
B:775(PRO)	−1.99	A:53(VAL)	−2.34	B:737(ALA)	−1.60	A:154(THR)	−0.80	B:755(GLY)	−0.80	B:888(GLU)	−0.90
B:758(MET)	−1.89	B:759(GLU)	−2.32	B:760(LEU)	−1.34	A:153(ALA)	−0.49	B:734(SER)	−0.79	B:805(TYR)	−0.89

**Table 2:** Affinity of molecular docking.

Peptide	Sequence	AKT1	JUN	CASP3	Average Affinity (kcal/mol)
		Affinity (kcal/mol)			
QE-8	QQLPQFEE	−6.70	−5.60	−6.10	−6.13
EQ-4	EEDQ	−6.20	−5.30	−6.40	−5.97
ER-4	EELR	−6.90	−5.30	−6.60	−6.27
LE-4	LSFE	−7.40	−5.60	−6.20	−6.40
YD-4	YTTD	−6.80	−6.30	−6.60	−6.57
YE-4	YTCE	−7.50	−5.80	−6.40	−6.57

## Data Availability

The original contributions presented in the study are included in the article/Supplementary Materials. Further inquiries can be directed to the corresponding author.

## Supplementary Materials

The following supporting information can be downloaded at: https://www.mdpi.com/article/10.3390/foods13233805/s1, Table S1. Prediction of umami peptides and disease targets.
