# Supplementary material for: Exploration of Bioactive Umami Peptides from Wheat Gluten: Umami Mechanism, Antioxidant Activity, and Potential Disease Target Sites
Source: Foods. 2024 Nov 26;13(23):3805. doi: 10.3390/foods13233805 (PMC11640069; doi:10.3390/foods13233805)
Supplement: Supplementary file 1 [file foods-13-03805-s001.zip › Table S1.pdf]

**Table S1.** Prediction of umami peptides and disease targets

| | | Oxidation-associated targets | | | | |
|---|---|---|---|---|---|---|
| **QE-8** | **LE-4** | **ER-4** | **YE-4** | **YD-4** | **EQ-4** | **Diseases** |
| EGFR | DLG4 | CFB | HMGCR | OPRM1 | CASP8 | POLG |
| SRC | HMGCR | HGFAC | OPRM1 | CASP1 | FOS | MGME1 |
| ACE2 | BACE1 | WDR5 | DLG4 | OPRD1 | CASP6 | NOL3 |
| PLG | HLA-A | FURIN | OPRD1 | IL1B | CASP7 | NUBPL |
| GSK3B | TACR2 | PCSK6 | ACE | XIAP | CASP1 | SOD2 |
| KIF5B | OPRD1 | NRP1 | ITGB1 | EDNRA | GZMB | SCO1 |
| DHODH | ACE | NTSR2 | HLA-A | RRM1 | HLA-A | FOXRED1 |
| ALAD | REN | NTSR1 | PDYN | PDYN | SLC5A1 | NOS2 |
| CTSD | OPRM1 | ADAM8 | CASP1 | CCKBR | CTSD | MPV17 |
| B3GAT1 | LCK | ITGA2B | MME | HLA-A | HLA-DRB3 | GFM1 |
| IGF1R | SRC | ITGAV | LCK | CALCRL | TNFRSF10A | ATP5F1D |
| F7 | ANPEP | MAPK8 | SRC | ITGA2B | ELANE | AIFM1 |
| PRDX5 | PTGS2 | DLG4 | ANPEP | CBX7 | CTSB | MTO1 |
| ME2 | CALCRL | ITGB1 | ITGB7 | TACR1 | XIAP | PNPT1 |
| PNPO | MME | SLC5A1 | STAT3 | HMGCR | DLG4 | MECP2 |
| FGF1 | CBX7 | F2 | RRM1 | OPRK1 | ITGB5 | NDUFA1 |
| CPB1 | CBX4 | F2RL1 | PRLR | MAPK1 | SPSB2 | PTCD3 |
| RNASE3 | ITGB1 | F10 | FNTA | NTSR1 | ADAM8 | MTFMT |
| GSTP1 | ITGA4 | TMPRSS6 | MAPK1 | STAT3 | REN | AARS2 |
| TREM1 | GALR1 | HPN | NTSR1 | ITGB1 | CTSE | LXN |
| EEA1 | GALR2 | CTSH | TACR2 | PIN1 | PGA5 | NOS3 |
| CA2 | EDNRA | ST14 | UTS2R | TACR2 | PRKCE | ELAC2 |
| TGM3 | CTSE | CTSL | ENPEP | DLG4 | CTSL | TP53 |
| PTPN1 | CASP1 | CAPN1 | MAOA | NTSR2 | ADAM9 | CARS2 |
| SNRPA | KLK1 | CTSB | CBX7 | YARS | BACE1 | FARS2 |
| TPI1 | CTSD | ITGB5 | TACR1 | SLC6A3 | ITGA2B | NOS1 |
| CMA1 | BACE2 | EPHX2 | ITGA2B | ACE | NPR1 | CAT |
| PAK6 | ITGB3 | HLA-A | LAP3 | ENPEP | NPR3 | SOD1 |
| RIDA | ITGA2B | DPP4 | NTSR2 | ITGA4 | GNPAT | ATM |
| PCK1 | RRM1 | CASP8 | OXTR | MMP1 | UBE2I | DDB2 |
| TTR | OPRK1 | FOS JUN | OPRK1 | HLA-DRB3 | ANPEP | TNF |
| OTC | PDYN | REN | TACR3 | MME | CASP3 | POLR1C |
| PDE4B | XIAP | CPB2 | REN | TACR3 | F2 | PARP1 |
| GLRX | FPR1 | CTSE | MMP12 | LAP3 | FURIN | DDB1 |
| CA1 | PRLR | PGA5 | ITGA4 | PTGS2 | PCSK6 | NARS2 |
| PSPH | NTSR1 | PRKCE | PTGS2 | CBX4 | PGC | TSFM |
| TYMS | MMP3 | ADAM9 | SLC6A3 | CASP3 | ENPEP | HMOX1 |
| CDK2 | TUBB1 | ACE | MMP10 | SLC15A1 | ACE | XPC |
| ARF1 | MMP8 | TPSAB1 | CALCRL | GALR1 | MAPK8 | OGG1 |

| | | | | | | |
|---|---|---|---|---|---|---|
| MAPKAP K2 | KDM1A | TPP2 | AVPR2 | GALR2 | DPP4 | BRCA1 |
| RNASE4 | TACR1 | PGC | AVPR1A | BACE2 | FPR2 | RAB33A |
| MTAP | NTSR2 | C3AR1 | FYN | HDAC3 | NOS2 | MTRFR |
| PLAU | F2 | BACE1 | ABL1 | HDAC6 | ITGAV | MPO |
| ADH5 | HLA-DRB3 | ITGAV | TUBB1 | HDAC2 | F10 | TUFM |
| PIM1 | MMP2 | MEN1 | HAGH | OXTR | CELA1 | MDC1 |
| PYGL | NPFFR1 | CTSD | DNMT1 | HDAC11 | CAPN1 | XPA |
| ANG | NPFFR2 | PTGS2 | DNMT3B | HDAC10 | ITGB1 | GADD45A |
| CTSG | FNTA | PLG | BACE1 | EPHX2 | SIRT1 | RMND1 |
| PLEKHA4 | EPHX2 | CDK2 | AKT1 | ITGAV | CASP2 | NFE2L2 |
| NMNAT1 | QRFPR | CTSK | XIAP | CHRM4 | HGFAC | EARS2 |
| MIF | OXTR | GLO1 | TYMS | CHRM2 | CFB | XDH |
| FDPS | LAP3 | MME | HLA-DRB3 | CHRM1 | MME | VARS2 |
| GSTT2B | CHRM4 | CASP3 | ITGA3 | CHRM3 | PLG | CYCS |
| LPA | CHRM3 | CASP6 | AVPR1B | BACE1 | BACE2 | OLR1 |
| FOLH1 | MMP12 | CASP7 | LGALS3 | SRC | PSMB2 | CHEK2 |
| FHIT | MMP1 | CASP1 | GART | IDO1 | PSMB1 | GSR |
| DHFR | GNPAT | GSTK1 | YARS | NPY4R | PSMB5 | CASP3 |
| CD1A | CCND1 | TACR1 | XPNPEP1 | MLNR | NRP1 | DDIT3 |
| APCS | CCNA2 | UBE2I | XPNPEP2 | NCOR2 | PTGS2 | APEX1 |
| CSNK2A1 | ACKR3 | PRLR | HPRT1 | FPR1 | WDR5 | IL6 |
| IMPDH1 | MMP13 | FPR2 | ECE1 | BDKRB1 | GLO1 | PCNA |
| PAPSS1 | ITGAV | ANPEP | CBX4 | SORT1 | NTSR1 | TXN |
| DAPK1 | ITGB5 | CCND1 | BACE2 | PRLR | SIRT2 | SIRT1 |
| ACP3 | TRHR | CCNA2 | PIN1 | LCK | FNTA | CPT2 |
| BMP7 | TACR3 | NPFFR1 | DOT1L | DNMT1 | GSTK1 | CDKN1A |
| RORA | STAT3 | NPFFR2 | CA12 | DNMT3B | NTSR2 | CHEK1 |
| CDK7 | PGC | ELANE | CA9 | GRB2 | CPT1A | XRCC1 |
| MMP13 | C3AR1 | GZMB | CA4 | AVPR2 | CPT2 | MAPK14 |
| PNP | PSMB8 | KDM1A | IL1B | PSMB9 | CA4 | HADHA |
| C8G | AVPR1A | XIAP | GALR1 | PSMB10 | MMP3 | IL1B |
| ALDOA | NPPA | SIRT1 | GALR2 | PSMB8 | MMP9 | ATR |
| FNTA | MAPK1 | NMUR2 | GRM2 | GSTP1 | MMP1 | IFNG |
| CHEK1 | GHRL | HLA-DRB3 | C3AR1 | GLO1 | MMP2 | GSTM1 |
| HGF | HDAC1 | MMP3 | PGGT1B | CASP6 | MMP8 | FASTKD2 |
| PMS2 | ITGB1 | MMP9 | SORT1 | CASP7 | PDF | PON1 |
| PLA2G2A | MMP9 | MMP1 | EDNRA | CASP8 | ECE1 | ACADVL |
| CCNA2 | BDKRB1 | MMP2 | EPHX2 | GRIA1 | CA12 | GTPBP3 |
| MAPK10 | CETP | MMP8 | BDKRB1 | CHRND | CA14 | G6PD |
| RXRA | MMP10 | PDF | KLK1 | HDAC8 | CA9 | MAPK1 |

| | | | | | | |
|---|---|---|---|---|---|---|
| F10 | CASP3 | ECE1 | ATIC | GART | XPNPEP1 | TXN2 |
| DTYMK | CAPN1 | NPR1 | CPA1 | CAPN1 | PEPD | BRCA2 |
| IMPDH2 | CHRM5 | NPR3 | MMP2 | KLK3 | XPNPEP2 | TARS2 |
| HAGH | CTRC | LAP3 | KMT2A | AKT1 | LAP3 | GSTP1 |
| EPHB4 | GBA | F11 | SUV39H1 | QRFPR | RRM1 | BCL2 |
| SORD | CELA1 | PLAU | INMT | HAGH | CALCRL | GFM2 |
| ALB | ITGA4 | TNFRSF10A | SMYD2 | LGALS3 | ITGAV | ACADM |
| MMP8 | CHRM1 | XPNPEP1 | EZH2 | F2 | PRKCD | MAPK8 |
| PSAP | GHSR | PEPD | EZH1 | CCND1 | VDR | H2AX |
| ARF4 | BIRC2 | XPNPEP2 | SETDB1 | CAPN2 | ITGA3 | HADHB |
| PAH | MC4R | PRSS1 | CARM1 | CCNA2 | MMP13 | ERCC2 |
| GSR | MC3R | CXCR4 | PRMT1 | CHRM5 | MMP10 | RAD51 |
| HMGCR | SORT1 | HLA-DRB1 | EHMT1 | MMP3 | MMP12 | ATP5F1A |
| HSP90AA1 | PGGT1B | FNTA FNTB | EHMT2 | MMP2 | EDNRA | NBN |
| BCAT2 | SIRT1 | SIRT2 | ADORA1 | MMP10 | PRSS1 | MDM2 |
| NMNAT3 | HDAC3 | TMPRSS11D | CXCR4 | MMP8 | CDK2 CCNA1 CCNA2 | APP |
| NCS1 | HDAC2 | NOS2 | CHRM4 | FNTA | ADAM17 | PTGS2 |
| MME | HDAC11 | SORT1 | CHRM2 | ANPEP | GBA | INS |
| PDE4D | HDAC10 | KLK1 | CHRM1 | MMP9 | FUCA1 | ALB |
| CTSK | ITGAV | PRMT1 | CHRM3 | TUBB1 | SLC1A1 | TGFB1 |
| AKR1B1 | CASP2 | DPP7 | PIK3CA | UGCG | PLAU | EDN1 |
| MAPK14 | CXCR4 | ITGA5 | FNTA | CDK4 | JUN | MIPEP |
| PDE3B | SOAT1 | CDK4 | ITGB3 | CDK2 | ITGAV | DDIT4 |
| DPP4 | ITGB6 | CDK2 | FNTB | FNTB | ITGB3 | MUTYH |
| FABP5 | FNTA | CCNA1 | ITGA4 | HDAC3 | ITGB3 | GADD45B |
| PDHB | FNTB | CCNA2 | ITGA4 | ITGB3 | ITGA5 | GADD45G |
| RHOA | ITGA5 | ITGAV | | ITGA4 | FNTB | TP53BP1 |
| ESR1 | CDK4 | ITGB3 | | ITGB3 | | GSTT1 |
| GSTM1 | CDK2 | ITGB3 | | | | CPT1A |
| PPARG | ITGB3 | | | | | CCL2 |
| PDE5A | ITGAV | | | | | AKT1 |
| SULT2B1 | | | | | | GPX1 |
| MAPK12 | | | | | | RPA1 |
| G6PD | | | | | | CRP |
| ITPKA | | | | | | MRPL44 |
| HSPA1A | | | | | | CYP1A1 |
| HSPA1B | | | | | | ACE |
| NR1H4 | | | | | | PARK7 |
| KDR | | | | | | NQO1 |
| FABP3 | | | | | | JUN |

| | |
|---|---|
| MMP12 | PRKDC |
| THRB | CP |
| CDA | NFKB1 |
| GSS | ABL1 |
| LCK | CYP2E1 |
| DDX39B | IL10 |
| PPARD | CASP9 |
| EPHA2 | GAPDH |
| SDS | CYP3A4 |
| SULT2A1 | BAX |
| LGALS7 | CXCL8 |
| LGALS7B | MRPS22 |
| TK1 | SLC25A20 |
| RAB9A | MAPT |
| RAN | NFS1 |
| IMPA1 | HBG2 |
| GALE | OXSR1 |
| BLVRB | TRIT1 |
| LCN2 | WRN |
| PARP1 | HMGB1 |
| KIT | APOE |
| IGF1 | OSGIN1 |
| MMP7 | FANCD2 |
| NNT | NDUFS4 |
| MMP3 | CD36 |
| ISG20 | PRKN |
| ATIC | POLRMT |
| DPEP1 | VCP |
| BST1 | ACADS |
| RFK | SLC25A26 |
| SULT1A1 | ERCC6 |
| HSD11B1 | FAS |
| RNASE2 | PRDX5 |
| PKLR | SFXN4 |
| RAB5A | LMNA |
| KYAT1 | PRKCD |
| ERI1 | NUDT1 |
| NDST1 | EPHX1 |
| CDC42 | MTHFR |
| MTHFD1 | MSRA |
| WARS1 | FOXO3 |
| F2 | VEGFA |
| CYP2C9 | HIF1A |
| PADI4 | HBB |

| | |
|---|---|
| AKT2 | CDK2 |
| CBR1 | PPARG |
| NME2 | CYBA |
| SELE | ACOX1 |
| CCL5 | MB |
| GLO1 | ERCC5 |
| GSTM2 | CYP1B1 |
| NT5M | ACADL |
| BIRC7 | POLH |
| INSR | RPA2 |
| RAC2 | DDIAS |
| SERPINA1 | C1QBP |
| GM2A | CAV1 |
| EIF4E | SNCA |
| DUT | MARS2 |
| C1S | CDKN2A |
| AHCY | PRORP |
| GSTA1 | CYP2D6 |
| TAP1 | HSP90AA1 |
| NOS2 | DDIT4L |
| CASP3 | ERCC4 |
| REG1A | QRSL1 |
| AKR1C3 | ICAM1 |
| TGFBR1 | DRAM1 |
| MDM2 | NEIL1 |
| Pfkfb1 | ACAD9 |
| PPCDC | PRDX2 |
| AKT1 | RAD17 |
| CTSS | NOS1AP |
| HADH | COX5A |
| ITK | CYP1A2 |
| ITGAL | AGT |
| LGALS2 | HSD17B4 |
| AMY2A | FOXO1 |
| CLK1 | GATB |
| ELANE | SP1 |
| RND3 | MRPS34 |
| HDAC8 | TLR4 |
| HSPA8 | ALDH2 |
| RAC1 | LYRM4 |
| GSTA3 | ATP5F1B |
| OAT | XRCC5 |
| ARSA | RAD50 |
| ARHGAP1 | ABCD1 |

| | |
|---|---|
| PDPK1 | STAT3 |
| MMP9 | VWF |
| ACE | MRPS16 |
| PPARA | EP300 |
| HRAS | RYR1 |
| BTK | CYBB |
| UCK2 | CBS |
| ADAM17 | NTHL1 |
| PRKACA | ERCC1 |
| ABO | PNKP |
| LYZ | DDI2 |
| GPI | ERCC3 |
| SEC14L2 | HSPA4 |
| ABO | FMO3 |
| KIF11 | CDK1 |
| FABP6 | FXN |
| JAK2 | RRM2B |
| GSTZ1 | E2F1 |
| HCK | MRE11 |
| RXRB | MT-CYB |
| GRB2 | PPARGC1A |
| CASP1 | KNG1 |
| DCK | MAPK3 |
| AR | PTEN |
| LGALS3 | PIK3CA |
| CBS | APOA1 |
| GNPDA1 | HRAS |
| FGFR1 | DRAM2 |
| SULT1E1 | TIMM22 |
| FKBP1A | HFE |
| REN | SIRT6 |
| LTA4H | RAD18 |
| AMY1A | POLB |
| AMY1B | MTOR |
| AMY1C | MAP3K5 |
| ARL5A | TOPBP1 |
| LDHB | BLVRB |
| CRABP2 | RAD9A |
| RAP2A | PML |
| RAF1 | IL1A |
| PTK2 | KEAP1 |
| FECH | MICOS13 |
| APAF1 | ESR1 |
| APRT | MYC |

| | |
|---|---|
| GCDH | SOD3 |
| FABP7 | TYR |
| MMP2 | HADH |
| MMP1 | NDUFS1 |
| SSE1 | ATRIP |
| BACE1 | TP73 |
| SETD7 | GPX3 |
| ACAT1 | DDI1 |
| RAB11A | CDC25C |
| RARA | EME2 |
| GART | UCP2 |
| CHIT1 | MT-ATP6 |
| GSTO1 | MT-ND1 |
| HINT1 | MT-CO1 |
| F11 | HSPB1 |
| NR1I2 | DNM1L |
| THRA | XRCC3 |
| MMP16 | FASLG |
| CAT | NSUN3 |
| SPR | APOB |
| GP1BA | TERT |
| GCK | PLA2G7 |
| HNF4G | CREB1 |
| HEXB | SERAC1 |
| VDR | CYP2C9 |
| PITPNA | VARS1 |
| FGG | PRDX1 |
| GLTP | ERCC8 |
| IVD | PSEN1 |
| HMOX1 | EGFR |
| SYK | VCAM1 |
| CANT1 | PLK1 |
| ADK | CCND1 |
| Arl5b | MRPS2 |
| DCPS | MRPS7 |
| MAPK1 | NOX4 |
| DCXR | SGCB |
| MAP2K1 | ATF2 |
| MAN1B1 | PARP2 |
| PIK3R1 | MSH2 |
| NR3C1 | CREBBP |
| STAT1 | PPARA |
| CTSB | MT-CO2 |
| HPRT1 | RBBP8 |

PINK1
DYNLL1
ECHS1
TRMT5
SHC1
HP
XRCC6
TAMM41
MFN2
MAPK10
CASP8
FANCA
TRMT10C
TREX1
RAD23B
AGTR1
BMP6
MRPL3
ALKBH1
ETFDH
PRDX6
TOP1
HSPA8
SLC25A4
FANCI
CDKN1B
MT-CO3
BRIP1
SLC17A5
FEN1
MIEF2
GPX4
MT-ND5
THBD
ALDH3A2
CTNNB1
MLH1
PTGS1
SQSTM1
SERPINE1
NLRP3
MT-ND4
DMD
IL18

OXR1
SRC
SETX
SDHB
MT-ND2
GSS
BCL2L1
ALKBH2
NDUFV1
RAD1
MRPL12
GCDH
SERPINA1
PIK3CG
SELE
MGMT
NEIL2
RPS27A
MAOB
RAD51C
CKB
ANXA5
LONP1
CYP2C19
GATC
PALB2
MIF
SDHA
GGT1
POR
GLRX
NOX1
IL17A
MC1R
NOSIP
RFWD3
KRIT1
FMO1
FOS
NPPA
REN
MT-ND3
GCH1
ADIPOQ

NOSTRIN
GCLC
ETFA
SELP
IGF1
P4HB
CS
MMP9
IRS1
EHHADH
RPS3
EPO
ADH5
TERF2
NDUFS2
BARD1
MTR
UCP3
S100B
TF
HPRT1
PRDX3
FANCC
POLK
CDC25A
RHOA
HUS1
BDNF
RB1
HLA-DRB1
CYP11B2
STAT1
DDAH2
TXNRD1
MAOA
LEP
INSR
MPG
SCP2
HSPA5
GPT
NDUFS8
MRPL39
FANCG

LDLR
APAF1
NDUFS3
PDE5A
TXNRD2
TOP2A
TH
RNF168
NDUFAF2
MUC1
KRAS
CUL4A
AARS1
ALDH3A1
UBC
MRPS25
TEFM
ALOX5
CRLS1
MAPK9
BLM
CKM
FN1
MSRB2
CHUK
SELENON
HSPA1A
CDK5
XRCC2
MAP2K1
MT-ND6
TLR2
JAK2
CDKN3
NPM1
RAD52
SMAD3
CYP2B6
MRPS23
GPX7
MBL2
EGR1
SLC22A5
AGER

HSD17B10
CFTR
PTPN11
SETD2
SUOX
FTO
HMOX2
MSRB1
HSPD1
ETFB
HIPK2
DDAH1
GFAP
MT-ATP8
S100A8
ELANE
CYP4F3
PPP1R15A
KDR
AKR1A1
MMP2
TRAF6
HDAC2
CCNB1
DNAH8
TP63
CYP3A5
RAC1
TPO
CTSD
ASL
CYGB
CRAT
PRKAA1
ABCC1
BID
BAK1
ALDH9A1
ENO2
GUCY1A1
MMP1
NDUFA2
HMGCR
RPA3

HDAC1
CFH
PRDX4
EDNRA
SMARCA4
NFKBIA
S100A9
DNMT1
FMO2
PON2
GSTM3
TNFRSF1A
HDAC9
GSTA1
PRKCB
BAD
NPY
PRKAA2
ARG2
MSH6
PAH
KAT5
NDUFS7
AOC3
EPRS1
LPO
PRNP
USP7
POLI
CASP1
ACP1
IL13
LPL
PGD
IRF1
MRPS28
CDK4
IL4
TNFRSF1B
OGDH
LRRK2
TTR
CYP2A6
TARDBP

BACE1
IKBKB
NDUFB9
AHSP
ARG1
UGT1A1
VIP
IKBKG
NAGLU
IL2
ADM
SPP1
BCKDHB
NDUFA10
IDH1
EPX
NPPB
EEF1A1
TRIM28
TRPM2
ATF4
GCK
NOTCH3
PRKG1
F2
SIRT3
NGF
AMBP
RANBP2
SPG7
PTPN1
CHAT
RECQL4
BRCC3
RAF1
H6PD
MTRR
APTX
OPA1
CALCA
PIK3R1
BMP2
SESN1
HLA-B

PARG
COX4I1
FOXM1
HTT
CSNK2A1
LIG3
DLG4
HBA1
MAPKAPK2
GLUL
HGF
SMARCAL1
ASS1
NDUFS6
NDUFA6
CYP19A1
CCL3
SMAD4
RETN
RBX1
ITGAM
VIM
FLT1
SMUG1
APOH
NDUFA9
DAXX
MT-ND4L
NAT2
PPP5C
CASP2
BRAF
MRPS14
NDUFB10
UBE2N
VDAC1
NDUFA11
AS3MT
NCF2
CYP27A1
PLA2G6
EGF
MAP2K4
LTF

ACACA
TUG1
ADRB2
NDUFV2
SYK
TIGAR
UVSSA
GCLM
MAD2L2
SLX4
TIMP1
TXNIP
BCS1L
TTN
BCL2L11
STUB1
FGF2
DLD
SCARB1
EIF2AK2
ALDH1A1
AKR1B1
ACTB
SLPI
AHR
SESN2
NOTCH1
CD40LG
YARS2
ADH1C
NDUFC2
PPIG
ADH1B
CHCHD4
CLEC4A
MCPH1
ADA
BIRC5
GSK3B
IL1RN
EPHX2
FMR1
HSF1
GBA1

UCP1
REV3L
SMC1A
CYP4F2
SMPD1
REV1
WT1
RIF1
RELA
ABCA1
ALKBH3
USP1
MSR1
ATP5F1E
LGALS3
THBS1
CYP11A1
FOXP3
POLGARF
FUS
LIG1
SLC1A3
EIF2S1
ARMT1
FTH1
PLAT
CUL4B
CCNA2
TFAM
PIK3C2A
H2BC21
ODC1
VDR
SIRT2
NDUFA12
GFER
PSMC6
GLA
HMGCL
SFN
CD40
APEX2
MBP
LTA

YWHAE
CDK7
ERBB2
SDHD
NGB
MCL1
NRF1
XRCC4
HNF4A
MLYCD
ATP5PO
CLU
CPOX
GLRX2
TNFSF10
UNG
CKMT2
NOD2
UBE2D3
ATG9B
MYD88
FBXL4
ALAD
TFRC
DNM2
USP28
FH
RECQL
MMP3
GDNF
GGA2
OXA1L
PRPF19
DCLRE1C
FBXW7
POLQ
MGST1
CTLA4
POLDIP2
MMUT
SLC25A3
TG
PC
USP10

PSMA6
ACO2
TWF2
SAMHD1
PPARD
NDUFAF1
INTS3
ISCU
PLA2G4A
PLK3
PLA2G2A
F5
CD46
STK11
CBX3
TDP1
EIF2AK3
COX15
UCHL1
KRT18
CYP2C8
NDRG1
ADH7
FDXR
LOX
DUSP1
UBB
CXCL10
CALR
ACACB
CCL4
TUBB3
CDC42
CCL5
PSMD3
CRYAB
IL1R1
PTPN3
PRKCA
CKMT1B
NOA1
AKR1C3
COMT
AOX1

PECAM1
KLF4
POLD1
MORC2
AR
TRPV1
VHL
SURF1
ENG
IDO1
ACO1
F3
HAO1
LYN
HERC2
GRIN2B
GJB2
TMEM126B
HSP90AB1
PLG
ANGPT2
TRPV4
MYH9
LCN2
SLC2A1
CYP24A1
FANCM
DECR1
WARS2
MME
ACHE
C11orf65
GRB2
NDUFA13
GADD45GIP1
MSRB3
FDX2
PEX5
ACAA1
AMACR
GHRL
BABAM2
PSMC4
YWHAQ

B2M
ETHE1
PTPN6
AGK
DNASE1
HBA2
ELN
TLR9
PAXIP1
GNAS
CD55
HNRNPK
GLO1
LDHA
CTNS
BMI1
CSF1
PMS2
CD44
RFC1
SLC1A2
JUNB
MDM4
CYP4A11
PSMA7
ACOX2
XIAP
NDUFA8
PIK3CB
ABCB1
PHYH
UBQLN4
IGF1R
ITGB1
HLA-DQB1
CPT1B
CYB5R3
SLC12A2
PDGFRB
NEIL3
MKI67
AKR1C1
GSTM2
NME1

COL2A1
FASN
TUBB
GJA1
SSBP1
GDAP1
TWNK
DES
SORD
CASP7
SOCS1
PPM1D
CEP164
YY1
SMG1
TMPO
COX10
ALOX12
PTPN22
AGTR2
IL12B
GSTO1
PTS
CETN2
CD4
CYC1
SLC2A4
MYCN
ACAD11
NCF1
ESR2
ITGB2
TDG
IDH2
CDK6
PKM
APEH
DNA2
PLCG2
UHRF1
MCM2
LPA
RIPK1
TYMP

ATG5
STING1
AHCY
CHCHD10
FGFR2
NDUFB11
CXCR4
GRN
C5AR1
MAP3K1
CDH1
TNFSF11
COX6B1
POMC
ANGPT1
PTK2B
ATP2A2
ACTA2
APOA2
TCF7L2
RNASE3
BIVM-ERCC5
VRK1
KDM1A
EDNRB
MET
CYP17A1
UQCRC1
RUNX2
H4C16
TOP3A
TGFBR2
H2AC20
H2AC4
ACTA1
ATP7B
H3C1
KARS1
CYB5A
NDUFB3
FCGR2A
MMP13
IFNA1
CHKA

PTPA
ACADSB
CD163
TKT
YWHAG
PVALB
TJP1
CAV3
FZR1
CEBPB
SLC7A1
PLAU
HSPG2
ABCD3
PIN1
PRIMPOL
BBC3
PRKAB1
CYP3A7
RAC2
HNF1A
FAM120A
NDUFB8
ADCYAP1
CHD1L
YWHAZ
IREB2
ACE2
NDUFAF5
SSRP1
NEFL
PSMC3
RRM1
CXCL12
RARA
MFN1
ACTG1
FANCB
PRTN3
FECH
ACAA2
SFPQ
UGT1A6
PRKCZ

TIMM50
HUWE1
UQCRC2
POLD3
FOSL1
NAMPT
GLUD1
FGF7
OTC
NR3C2
SLC11A1
CAPN1
ATF3
CSF3
CYP20A1
NDUFB7
F8
CSF1R
CYP2U1
GSTA4
TYMS
DNAH9
IRAK1
SMAD2
SCD
ELAVL1
H4C1
TGFB2
MDH2
SLC6A3
PSMC5
CYP7B1
HLTF
GPX2
TLR3
COX6A1
LIPE
DHX9
ADD1
CASP6
TALDO1
PTGIS
ANXA2
UBE2I

CCL11
CCN2
CYP2J2
PSMD4
NTRK1
SUCLA2
NRAS
ACOX3
TRAF2
TAFAZZIN
SLC25A5
NR3C1
CTSB
GAL
TMEM161A
UBE2A
KCNJ11
GPX5
DHFR
NR1H4
CDC25B
KIT
SLFN11
DTL
IL5
SMARCA5
VCL
FTL
ALOX15
GTF2H5
ATAD5
SNTA1
CHD4
SST
MTLN
STK25
TERF1
PARP9
LARS2
COX6A2
ENO1
GSTA2
CAMK2G
NABP2

PDIA2
KCNJ5
PSMD1
DARS2
DSPP
CNTF
CALM1
GUSB
SUMO1
SRXN1
MUS81
SLC7A11
CGAS
IRS2
CTSG
PDIA3
GZMB
LIG4
PRKCG
YAP1
SERPINF1
TAOK1
FANCE
AKT2
IGHMBP2
TINF2
COX8A
ABCB7
PKD1
IKBKE
CSF2
TMEM67
NDUFAF3
COL4A4
BDKRB2
BNIP3
RUVBL2
HELLS
PSMD6
TAC1
PPIA
HBEGF
RRM2
UBE2B

BRD4
PXDN
FGB
ENDOG
PSMD2
RAD23A
COPS5
ETS1
LCAT
ATP1A1
BANF1
KCNMA1
PHB1
UQCRFS1
SMC3
FANCF
SPAST
PAK1
PSAP
CEL
UQCRQ
AURKA
SPR
IL15
ATXN3
BCKDHA
ADAR
IL3
CCAR2
CRH
ERO1A
CYP7A1
GRIN1
UBA1
TIMELESS
DUOX2
APC
LYRM7
ITGB3
CYP46A1
PDHA1
SULT1A3
EEF2
PDGFRA

TNFAIP3
PSMD11
NR2C2
MICAL1
ADAMTS13
TSPO
NDUFV3
IFNB1
NR1H2
COQ2
SHEP2
AT
FRDA
ALD
DJ1
ARMD1
FOXO3A
BHLHE40
ABCD2
COQ8A
UVSS3
EAOH
MSA1
SLC13A3
COXPD6
PD
WND
ALS1
GSSD
CUX2
RECQL2
COXPD28
COXPD39
MAGEE1
TP53INP1
IQCB1
PDRG1
SPRTN
NORAD
PANDAR
TMEM150B
SGK1
SDHC
PPIF

HMCES
HPR
RPRGL1
FECD1
NBIA3
SPG81
FLD1
NDUFA5
ATG4D
SELENOI
FTMT
DNAJC30
COXPD10
COXPD52
COXPD53
COXPD36
COXPD1
COXPD2
COXPD3
COXPD4
COXPD5
COXPD7
COXPD8
COXPD9
COXPD11
COXPD12
COXPD13
COXPD14
COXPD15
COXPD16
COXPD17
COXPD18
COXPD19
COXPD20
COXPD21
COXPD22
COXPD23
COXPD24
COXPD25
PNSED
COXPD27
COXPD29
COXPD30
COXPD31

COXPD32
COXPD33
COXPD34
COXPD35
COXPD37
COXPD38
COXPD40
COXPD41
COXPD42
COXPD43
COXPD44
COXPD45
COXPD46
COXPD47
COXPD48
COXPD49
COXPD50
COXPD51
COXPD54
COXPD55
HUMOP2
COXPD56
COXPD57
COXPD58
COXPD59
HUMOP1
OSGIN2
HSD17B6
UVSS2
ATOX1
PRXL2C
TTPA
NFE2L1
SELENOP
UBIAD1
PRXL2A
MTTP
CETP
ATF1
BACH1
MT3
GABPA
SLC22A12
NFE2L3

CUL3
PON3
SLC22A4
JUND
AIFM2
IL2RA
BGLAP
RNF7
MAF
MAFG
ARMS2
MAFK
SELENOW
IGFBP3
MPV17L
SLC4A11
NQO2
CXCL1
APOA4
PSMA3
SLC23A2
BMP7
LANCL1
CSN1S1
HPX
SLC11A2
PEPD
KL
BLVRA
BCHE
FABP1
MAPK7
NR1I2
CFLAR
CD79A
ALPP
TGFA
SLC23A1
ABCC2
TRIM21
ABHD10
NTF4
MT2A
TIMP2

CTH
SEC14L2
SERPINB1
HPGD
GLS2
IVL
NKRF
MT1A
PGF
PTK2
SLC2A9
FGF21
ABCG2
PRL
RBP4
CSNK2B
ST3GAL4
IGF2
ITIH4
PSIP1
NBR1
AKR1C2
TBC1D24
APOM
PSMA4
PGK1
ANPEP
NTF3
IL12A
MTF1
LBR
RABEP2
GAST
ELK1
NOX5
IL24
BTG2
TNFRSF10B
LIAS
LPXN
VEGFD
ALDH18A1
FOSB
PLCG1

FLG
SIRT5
TAT
STS
SECISBP2
PSMA2
MUC2
PDGFA
TGIF1
NTRK2
IL4R
SELENOK
MYO3B
VEGFC
DRD2
COQ9
CTRL
MAP2K6
COQ10A
FDX1
NTS
UGT1A10
KLK3
BMAL1
IL6R
LCN1
MAP3K7
COG2
BMP4
TNFSF9
ERG
AMH
SHBG
AKR1B10
HRH2
ENOX2
DGKQ
NFE2
MT1E
CD28
MPST
MAP2K3
CXCL2
IGHE

BIRC3
ARSH
PLD1
REG3A
PEDS1
CCL20
DIO2
OPTN
SLC52A2
PPARGC1B
BMPR2
PRKCE
GLS
KLF2
PPOX
SRF
SELENOM
APOC3
TNFRSF11B
PDGFB
NFKB2
SGSH
CGB5
GLRX3
BOK
KITLG
IL18R1
CFL1
IFI27
RACK1
CCK
LALBA
ACAN
DPAGT1
AFP
SLC25A10
SERPINB2
THPO
RHOD
DHCR24
S100A7
TPPP3
VKORC1L1
SELENOS

LIF
CD14
SIK2
CDH5
FGF19
CSN2
CLDN5
TGFBR1
IL2RB
TNFRSF11A
GH1
SLC2A14
PPP2R1B
METAP2
ADAMTS4
PPP1R12A
MLLT3
SLC30A5
HSPA14
DDX18
LST1
CYP4F11
IFRD2
TET2
PAM
IBSP
INTS2
PRMT1
SLC2A3
RORA
COQ6
KIF12
PRXL2B
HSPB2
TGFB3
PRKACA
UTS2
TULP1
GDF15
ALOXE3
SLC1A1
FOSL2
NME5
FOXR1

SMN1
PTX3
KLF10
CXCL16
CXCR6
S100A6
PIAS3
SLC25A30
ABCG1
NCF4
PLD2
AREG
CXCR2
PCBD1
SMOX
IL23A
IL11
CREB3
GATA4
STAT5A
IGFBP1
CA3
ARNT
BCAR1
BTK
CCR5
NGFR
TNFRSF10A
GPLD1
NFKBIB
IPO11
SULT1A1
COL1A1
FYN
IL1RAPL2
STAT6
DIABLO
F2RL1
TRAF4
TEK
MDK
UBE2E2
TNNT2
S100A4

RGN
ABCC3
PEBP1
FDFT1
PRKCH
NRG1
MYH6
TNFSF13B
CXCR1
OSM
PF4
IL19
NFKBIE
SREBF1
HAGH
TNFRSF10C
UCN
PCK2
G6PC1
GSTZ1
FLT4
PGR
GAMT
SLC26A4
NTSR1
SLC25A14
MMP19
CEBPG
COQ10B
ERBB3
SI
PROS1
ESRRA
PIR
MAT1A
MAT2A
HAL
RBM3
TPH1
KCNH2
ERN1
LMNB1
DGKE
SET

OLA1
UBE2D2
BCLAF1
ALDOC
KLRK1
LRP2
GABARAP
POU2F1
BLZF1
LEPR
HSPA9
ARSB
HLA-DRA
PFKFB3
RCAN1
PPP1R13L
DPP3
PAEP
RPE
AKR7A2
IL17RA
PLA2G1B
TNFRSF10D
GNRH1
IL16
CCL17
MYO1A
PLTP
ACAT1
SRD5A1
GGCX
KRT1
CD38
HRH1
KHK
AMPD3
LIPG
SYVN1
NIBAN2
GABPB1
FGF1
SLC2A10
UBE2E3
TSC22D3

AMN
GNAI2
ADORA3
CHGA
MAP2
NME2
PABPN1
SMAD7
FADS2
NUMB
S100A11
SELENBP1
PLK2
CEBPD
CARTPT
ERP44
GMFB
GIP
KPNA6
TXNDC5
TXNL1
TXNDC12
ZDHHC3
SLC39A2
MT1G
RBM45
BCL2L2-
PABPN1
IGF2R
TNFRSF8
CCL27
TXNRD3
CHRNA3
SOAT1
ESRRB
NNT
RNF2
LIPC
PPA1
UGT1A9
DSP
ATP1A2
GPD2
CHI3L1

SORT1
HMBS
GREM1
OCLN
TAC3
ASPA
HAVCR1
PREP
MYBL2
PKP1
VSNL1
GSTA3
TPPP
CBR3
DIP2A
ULBP2
CSN3
DCTD
NXNL1
PAQR3
CACUL1
FLNA
MYH7
NRP1
IL10RA
IL7
PPBP
RAC3
TIMP3
VEGFB
CYB5R1
CXCL6
MAP3K13
TMEM241
SFTPB
EHMT2
MTATP6
POAG
NF1
HD
PITX2
ARCL3A
EPRPDC
FCAS2

| |
|---|
| EPAS1 |
| VNN1 |
| NLRP12 |
| ERO1L |
| ATP5PD |
| T2D |
| WBS |
| HGPS |
| KTCN1 |
| FHCL1 |
| AML |
| CMYP3 |
| PSAB |
| RP4 |
| IMDDHH |
| PEBEL2 |
| THMA3 |
| PCOS1 |
| COPD |
| ARHI1 |
| MS4 |
| CCND3 |
| TET1 |
| CYRIB |
| RHO |
| SLC16A1 |
| FOXO4 |
| SHOX |
| COQ7 |
| SCARA3 |
| ERVW1 |
| STIM1 |
| CTSV |
| KMT2B |
| GDPD5 |
| POMP |
| PGAM5 |
| ERCC6L2 |
| TXNDC17 |
| FAM213A |
| TRNAU1AP |
